# Collagen fibril formation at the plasma membrane occurs independently from collagen secretion

**DOI:** 10.12688/wellcomeopenres.23776.1

**Published:** 2025-08-29

**Authors:** Adam Pickard, Richa Garva, Antony Adamson, Ben C. Calverley, Anna Hoyle, Christina E. Hayward, David Spiller, Yinhui Lu, Nigel Hodson, Oriana Mandolfo, Kevin Kim, George Bou-Gharios, Joe Swift, Brian Bigger, Karl E. Kadler

**Affiliations:** 1 School of Biological Sciences, Wellcome Centre for Cell-Matrix Research, The University of Manchester, Manchester, England, M13 9PT, UK; 2 Medicine and Health, Manchester Academic Health Science Centre, The University of Manchester, Manchester, England, M13 9PT, UK; 3 Pulmonary and Critical Care Medicine, University of Michigan Medical School, Ann Arbor, Michigan, USA; 4 Ageing and Chronic Disease, University of Liverpool Institute of Life Course and Medical Sciences, Liverpool, England, L7 8TX, UK; 5 Insitute of Regeneration and Repair, The University of Edinburgh Edinburgh Medical School, Edinburgh, Scotland, EH16 4UU, UK

**Keywords:** assembly, collagen; exocytic, extracellular matrix; fibroblasts; lysosomal storage, mucopolysaccharidosis, protein trafficking; secretion; SILAC, translation.

## Abstract

**Background:**

Collagen fibrils are the primary supporting scaffolds of vertebrate tissues, but the mechanism of assembly is unclear.

**Methods:**

Here, using CRISPR-tagging of type I collagen, high-resolution light imaging, and SILAC labelling, we elucidated the cellular mechanism underlying the spatiotemporal assembly of collagen fibrils in cultured fibroblasts.

**Results:**

Our findings reveal the multifaceted trafficking of collagen, including constitutive secretion, intracellular pooling, and plasma membrane-directed fibrillogenesis. Notably, we differentiated the processes of collagen secretion and fibril assembly and identified the crucial involvement of endocytosis in the regulation of fibril formation. By employing Col1a1 knockout fibroblasts, we demonstrated the incorporation of exogenous collagen into the nucleation sites at the plasma membrane through these recycling mechanisms.

**Conclusions:**

Our study sheds light on a complex and previously unidentified collagen assembly process and its regulation of health and disease. Mass spectrometry data were available via ProteomeXchange with the identifier PXD036794.

## Plain language summary

Fibrous tissue such as tendons, ligaments, skin and even the heart, contain millimetre long fibres that resist pulling forces and help maintain the shape and form of the tissue during everyday activities. The fibres are made of collagen protein molecules – by analogy, the collagen molecules are the building bricks in a wall or the steel rods in reinforced concrete. Studies of collagen fibres provide insights into a wide-spectrum of diseases associated with ageing and tissue degeneration. The fibres were seen using electron microscopes over 80 years ago but how they are formed was unknown, with several theories being proposed. In this study we tagged collagen molecules with probes that can be seen with light microscopes and observed, in real time, cells producing collagen and assembling the molecules into the fibres. We could visualise collagen molecules being synthesised within cells and being trafficked to the outer membrane (the plasma membrane) where the fibrils grew out from the cells and into the extracellular space. Therefore, the fibres grow on the surfaces of cells. We discovered that cells do not produce a continuous stream of collagen fibres but instead produce an intracellular pool of collagen that is ‘emptied’ on a rhythmic basis to make individual fibres. We looked at cells from people with mucopolysaccharidosis (a lysosome storage disorder) and showed that these cells were partially defective in starting new fibrils. This hints to how cells make the fibres, which could help medical scientists produce collagen-trafficking strategies to treat disease.

## Introduction

Collagen is the most abundant structural protein in mammals, accounting for approximately 25% of their total body protein mass ( Smejkal & Fitzgerald, 2017). It occurs mostly as *D*-periodic (where *D* is ~ 67 nm) fibrils that can be centimeters in length ( Craig *et al.*, 1989), ranging in diameter from ~12 nm to ~300 nm depending on the tissue and stage of development ( Parry *et al.*, 1978), and provides sites of attachment for a wide range of macromolecules, including signaling complexes and cell receptors ( Wickstrom & Fassler, 2011). However, elucidating the complex process of collagen fibril assembly remains challenging because of technical constraints. For example, individual fibrils are too narrow to be visualized by conventional light microscopy, and electron microscopy provides information only on preformed fibrils. Studies *in vitro* using purified collagen in the absence of cells showed that fibrils formed by self-assembly ( Gross & Kirk, 1958; Kadler *et al.*, 1987; Kadler *et al.*, 1990) but the *in vivo* mechanisms that determine the site of fibril assembly (nucleation) and how fibrils elongate remain elusive. *In vivo* observations using electron microscopy of embryonic tendons and corneas have revealed fibrils attached to plasma membranes ( Birk & Trelstad, 1984; Birk & Trelstad, 1986; Canty *et al.*, 2004; Kalson *et al.*, 2013; Starborg *et al.*, 2013; Trelstad & Hayashi, 1979; Young *et al.*, 2014). The studies described here were motivated by the need to record, in real time, fibril assembly in the presence of cells. We aimed to capture time-lapse images of collagen molecules as they trafficked through the cell and subsequently appeared as fibrils in the extracellular space.

In this study, we used CRISPR/Cas9 gene editing to tag collagen precursor proteins with photoswitchable and bioluminescent markers and used advanced imaging techniques to visualize fibril assembly at the plasma membrane. We further investigated the role of endocytic collagen recycling during the early stages of fibril nucleation. Here, we discuss our findings in the context of tissue engineering and therapeutic interventions targeting collagen-related disorders.

## Methods

### Mice

The care and use of all mice in this study were carried out in accordance with the UK Home Office regulations, UK Animals (Scientific Procedures) Act of 1986, under the Home Office Licence (#70/8858 or I045CA465). Permission included generation of conditional knockout animals. Col1a1 ^F/F^ mice were crossed with Col1a2-ER ^T2^ mice to generate the tamoxifen-inducible Col1a2-ER ^T2^:: Col1a1 ^F/F^ strain. Col1a1F/F mice were generated at the University of Michigan in accordance with the Institutional Animal Care and Use Committee (IACUC) protocol numbers PRO00012246 and PRO00010565. Ten-week-old Col1a2-ER ^T2^:: Col1a1 ^F/F^ mice were treated with tamoxifen by intraperitoneal injection according to the approved UK Home Office regulations (Project licence number I045CA465; Personal licence number P08B76E2B). Mice were humanely sacrificed using a schedule 1 procedure (CO _2_ overdose followed by cervical dislocation) by experienced personnel at the animal facility.

### Cell culture and treatments

Human cells were obtained and used for this study under the ethical approval REC08H101063, approved by the Northwest Research Ethical Committee, UK. Immortalized mouse embryonic fibroblasts, NIH3T3, were maintained in DMEM supplemented with 10% newborn bovine calf serum, penicillin, and streptomycin. The preosteoblast cell line MC3T3 was maintained in alpha modified MEM supplemented with 10% fetal bovine serum, penicillin, and streptomycin. Mouse tendon fibroblasts were cultured in DMEM:F-12 (1:1) medium supplemented with 5% L-glutamine, 10% dialyzed fetal bovine serum (FBS) and 10,000 U ml−1 penicillin/streptomycin) and at 37 °C with 5% CO2. For imaging, cells were grown on an ibitreat µ-Dish (Ibidi, Germany). NIH3T3 and MC3T3 cell lines were cultured with 20 and 200 µg/mL l-ascorbic acid, respectively, to induce collagen fibril formation. Immortalized tail tendon fibroblasts were generated as previously described ( Chang *et al.*, 2020; Pickard *et al.*, 2019) and maintained in a DMEM/F12 1:1 mixture supplemented with 10% FBS, penicillin, and streptomycin. The following concentrations were used: bafilomycin A (100 nM), brefeldin A 100nM, monensin (1 µM), chloroquine (1 µM), and ionomycin (10 µM). Cell survival was performed using Prestoblue blue (Thermo Fisher) after 72 h. Fluorescence measurements were collected on a Synergy Neo2 Multi-Mode Reader (Biotek) at excitation: 555/20 nm, emission: 596/20 nm (xenon flash, lamp energy low, gain 100 and read height 4.5 mm, 10 measurements per data point). For siRNA treatments, 200,000 cells were seeded in 6 well plates overnight and then transfected with 100 pmol siRNAusing RNAiMAX. Protein, RNA, and coverslips were harvested. Plasmid transfections were performed using Fugene 6 with 1 µg plasmid and 3:1 (Fugene 6:DNA) ratio. BFP-KDEL was a gift from Gia Voeltz (Addgene plasmid # 49150) ( Friedman *et al.*, 2011). HP47-BFP-RDEL was generated using PCR products of HP47 (PCR amplified from plasmid MC202692 (Origene)) and BFP-RDEL (PCR amplified from BFP-KDEL plasmid) using the primers detailed in Extended data 16. These were then assembled into a pLenti CMV V5-LUC Blast (w567-1) digested with BstXI (a gift from Eric Campeau (Addgene plasmid # 21474). Cy3 labell ( Doyle, 2018) Cy3 collagen was added directly to the medium at the indicated concentrations and immediately mixed.

### CRISPR/Cas9 knock-in design and vector construction

We used CRISPR/Cas9 to generate a Col1A2-Dendra2 knock-in cell line. Using the Sanger CRISPR design web tool, we selected a guide RNA ( *ACTTACATTGGCATGTTGCT* AGG) targeting exon 1 of the genomic sequence to generate a double-strand break immediately after the sequence encoding the signal peptide. The guide was delivered as RNA oligos (Integrated DNA Technologies, Coralville, US) in complex with Cas9 protein. A double-stranded DNA repair template was generated by PCR amplification of the 5’ and 3’ homology arms (800 bp each) of mouse genomic DNA. The Dendra2 mouse optimized coding sequence with a flexible linker sequence was synthesized (Genscript, US) and assembled using Gibson Assembly (NEB); primers are shown in Extended data 16. The knockdown of nanoluciferase to Col1a2 been previously described ( Calverley *et al.*, 2020).

To knock-in split nanoluciferase (Hibit) into mouse *Col1a2* exon 6, we used the gRNA *GCTGCTCAGTATTCTGACAA*. For human *COL1A2* exon 1 knock-in of Hibit, we used the gRNA *CAAGCTGAAGGCACTTACAT*. The repair templates for CRISPR-mediated knock-in of Hibit are detailed in Extended data 16. Briefly, the two primer sequences were annealed to generate hybrid ssDNA:dsDNA repair templates with 29 bp homology arms.

### CRISPR/Cas9 delivery

For knock-in of Dendra2, NIH3T3 and MC3T3 cells were seeded 24 h prior to transfection of the repair template, using Fugene 6 (Promega) at a ratio of 3 µL per 1 µg DNA. Cells were maintained in transfection mixture overnight and then fresh medium was added for approximately 6 h. Next, the cells were transfected overnight with 100 pmol of tracrRNA and crRNA complexed with 4 pmol recombinant cas9 protein using RNAiMAX (Thermo Fisher). The medium was then replaced, and cells were grown for 48 h before fluorescence and sorting were assessed. 

The sequences for Hibit knock-in crRNA purchased from IDT are shown in Extended data 17. 0.25 µL of crRNA (100 µM) was annealed with equimolar amounts of tracRNA (IDT) and complexed with Cas9 (1.5 µL 1 µM, NEB). CRISPR/Cas9 complexes and 100 pmol annealed repair templates were electroporated (Neon Transfection System, Thermo Fisher Scientific). The electroporation conditions for NIH3T3 were 1400 V, 20 ms, and 2 pulses using 10 µL tips and 50,000 cells, and for HFFs were 1700 V, 30 ms, and 1 pulse using 10 µL tips and 100,000 cells. Electroporated cells were grown in 12 well plates. 

### CRISPR/Cas9 validation

Dendra2 positive populations were sorted by FACS and grown to a sufficient number for editing validation. The cells were then sorted into single-cell clones. All assays and imaging were performed using single-cell-sorted populations. Individual cells were grown to a sufficient number for validation. Primers for the validation of the knock-in into genomic DNA (PR1 and PR2) were as follows: F-GGCAAGGGCGAGAGAGG and R-TTTTCTCCGACAGATTAGAGGGC. Real time validation of Dendra2::Col1a2 transcripts were assessed in single cell clones treated with 2.5 ng/mL TGF-b1 for 48 h using the primers indicated in Extended data 18, real time PCR was normalized to the geometric mean of Rplp0, Gapdh and Actb. The dendra2-col1a2 transcript sequence was validated by Sanger sequencing. Western blotting was performed on 4–12% tris-glycine gels with 25 µg cell lysate. Antibodies used in this study were collagen (Gentaur, OARA02579, dilution 1:2000 (WB), 1:500 (IF)), Dendra2 (Origene, TA180094, dilution 1:500), GAPDH (Sigma, G8795, dilution 1:10,000), Calreticulin (Stressgen; SPA-601, dilution 1:1000), Lamp1 (Santa Cruz, sc-20011, 1:500), vinculin (Chemicon; CBL233, 1:2000), Hp47 (Santa Cruz, sc-398579, 1:1000 (WB), 1:500 (IF)), and syntaxin 5 (Santa Cruz, sc-365124, 1:500(WB)) PDI (Abcam, ab180993, dilution 1:500 (IF)).

### Imaging

For the immunofluorescence detection of cellular and extracellular proteins, cells grown on coverslips were fixed with 4% paraformaldehyde for 20 min at room temperature and washed with PBS before permeabilization with 0.2% triton-x-100 in 10%FBS for 15 min. The antibodies used in this study have been described in detail above. 

**Figure 1.**
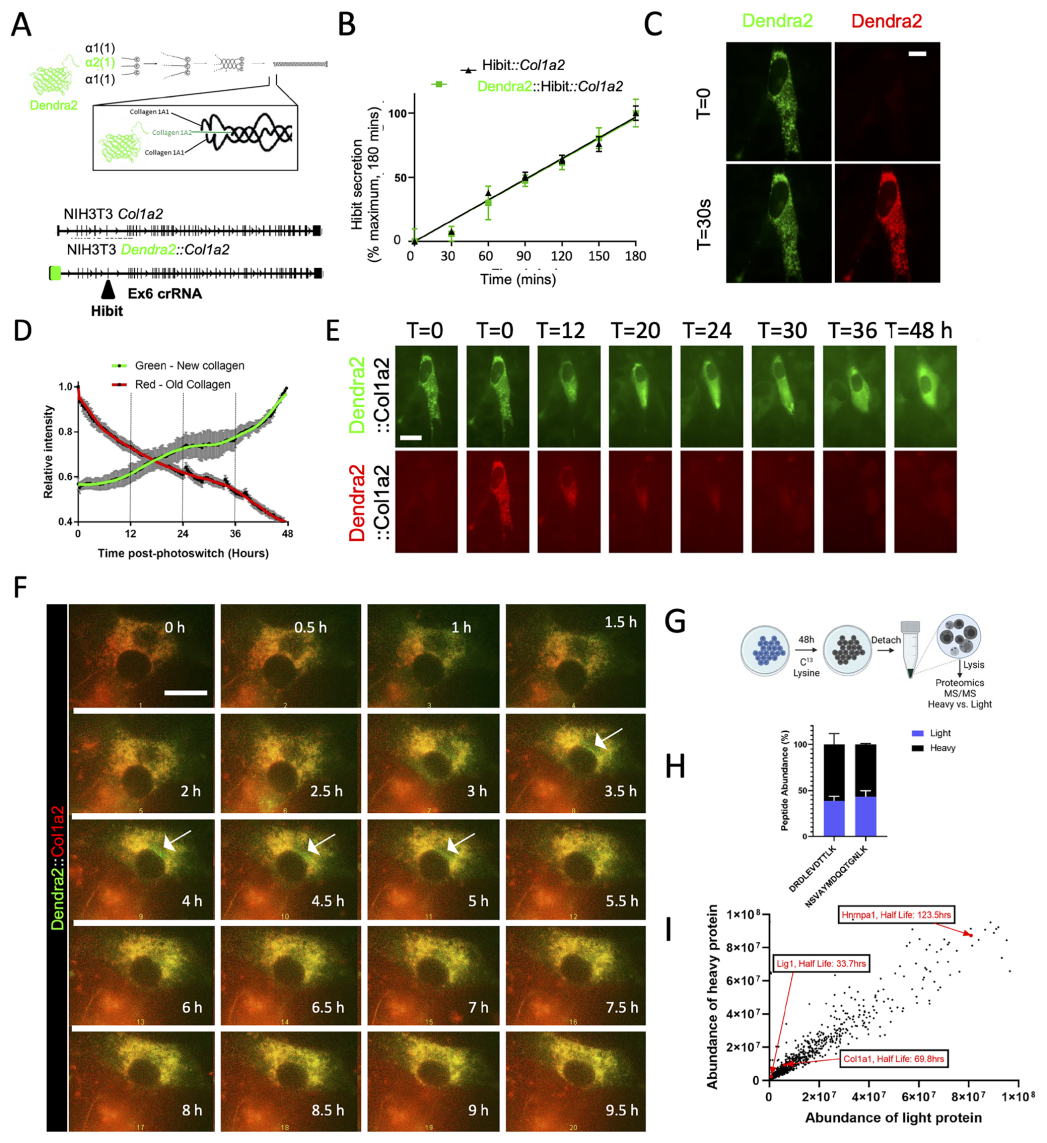
PCI secretion and collagen fibril assembly are separate processes. **A**) Schematic showing the site of integration of photoswitchable Dendra2 at the N-terminus of proa2(I) and the location of Hibit tagging sites in Col1a2. **B**) Introduction of the split nanoluciferase sequence encoding HiBit was confirmed by detection of HiBit in the medium of the edited cells. The rates of secretion of HiBit::Col1a2 and Dendra2::Hibit::Col1a2 were comparable. **C**) Photoswitching (green to red) of Dendra2-PCI after 30 s of exposure to 405 nm light. **D**) Time-lapse microscopy showing continued synthesis of Dendra2-PCI (green) and secretion of Dendra2-PCI (red). **E**) Measurement of fluorescence intensity in the green and red channels shows the synthesis (green channel) and secretion (red channel) of Dendra2-PCI over a period of 48 h. Note the 24 h rhythmic synthesis of Dendra2-PCI. **F**) Close-up showing newly synthesized Dendra2-PCI (green) transiting through a pool of Dendra1-PCI, which remained detectable beyond 9 h post-photoswitching. **G**) Schematic showing SILAC labelling of unedited NIH3T3 cells. The cells were incubated with C ^13^ lysine for 48 h before detachment, lysis, and proteomic analysis. **H**) Quantification of both heavy- and light labelled Col1a1-derived peptides demonstrates that not all PCI are secreted within 48 h. Peptide sequences shown are from the NC1 domain of proa1(I) mouse. N = 3 independent experiments. **I**) Global protein abundance in SILAC labelled NIH3T3 cells and the indicated protein half-lives were estimated by measuring the abundance of both heavy and light peptides for each protein, as described in Methods.

**Figure 2.**
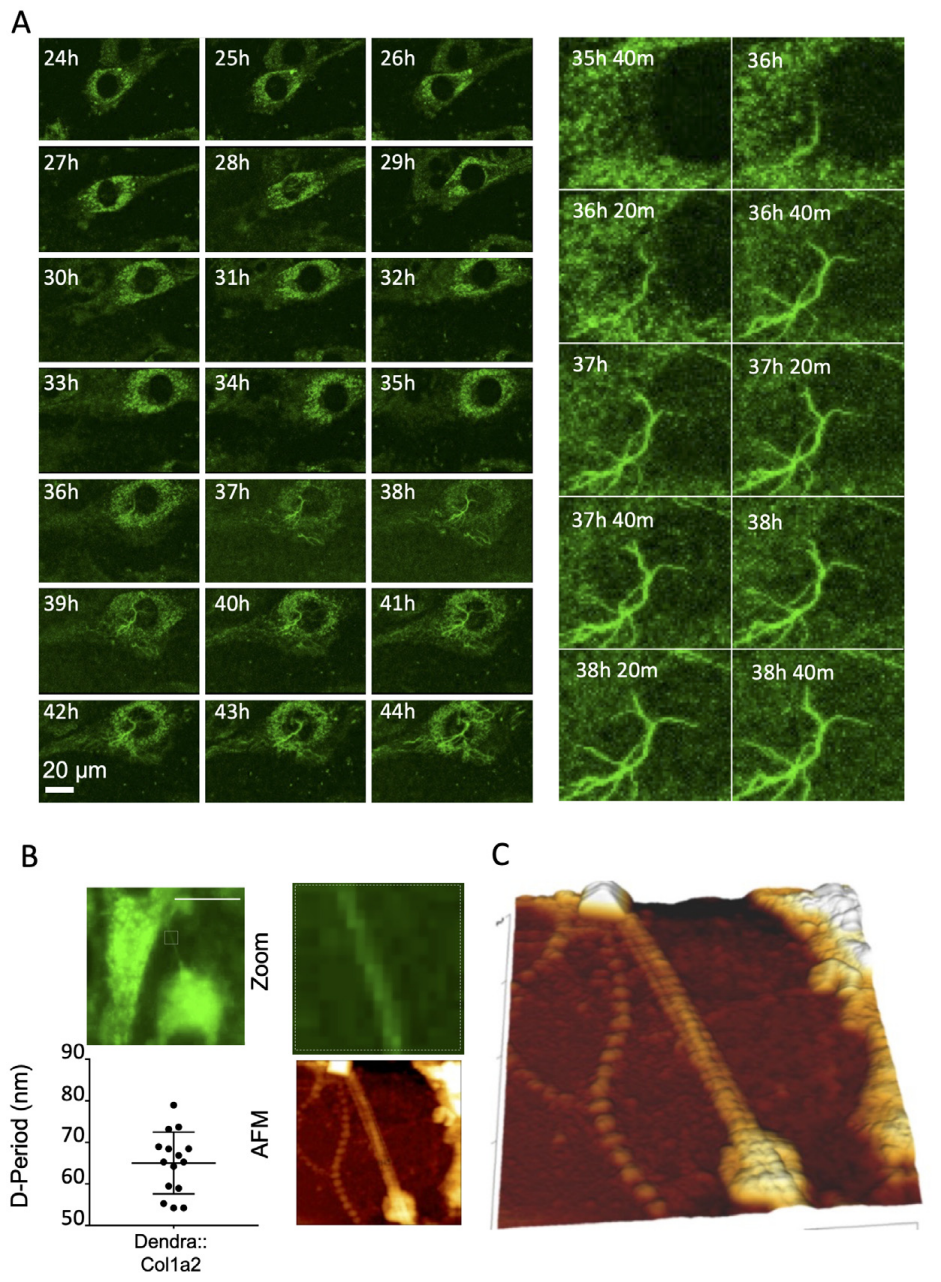
Collagen fibrillogenesis occurs on the plasma membrane. **A**) Time-lapse Airyscan microscopy of Dendra2::Col1a2 NIH3T3 cells. Dendra2 positive fibrillar structures were detected after 36 hours of culture. **B**) Dendra2::Col1a2 NIH3T3 cells were grown on correlative grid coverslips and imaged using a widefield fluorescence microscope. The samples were then fixed in order to perform AFM in the same region. Assessment of the periodicity of the Dendra2 positive fibril demonstrated a D-period of 65 nm. **C**) Zoom of the fibril shown in B. Scale bar 10 µm.

Figures 1C, E and F, Extended data 9, Figure 2B were acquired on an Eclipse Ti inverted microscope (Nikon) using a 60x objective, the Nikon filter sets for GFP and mCherry and LED (Lumencor) fluorescent light sources each with 300 ms exposure. Photoswitching was performed using a 30s exposure to UV LED light source (400 nm). The images were collected using a Retiga R6 (Q-Imaging) camera, and captured using NIS Elements AR.46.00.0 software. Pixel intensity was analysed using FIJI ImageJ ( http://imagej.net/Fiji/Downloads). Cells were maintained at 37 °C and 5% CO _2_. 

Images from Extended data 2A and 5C were collected on a Zeiss Axioimager D2 upright microscope using a 100x objective and captured using a Coolsnap HQ2 camera (Photometrics) using Micromanager software v1.4.23. Following 4% PFA fixation, the cells were permeabilized and stained with antibodies against Dendra2 (dilution 1:100) and collagen (dilution 1:200), and the secondary antibodies used were goat-anti-mouse488 (Cell Signaling; 4408, dilution 1:400) and goat anti-rabbit-Cy5 (Invitrogen; A10523, dilution 1:400). Specific bandpass filter sets for FITC and Cy5 were used to prevent bleeding from one channel to the next. All images were processed and analyzed using the Fiji software.

**Figure 3.**
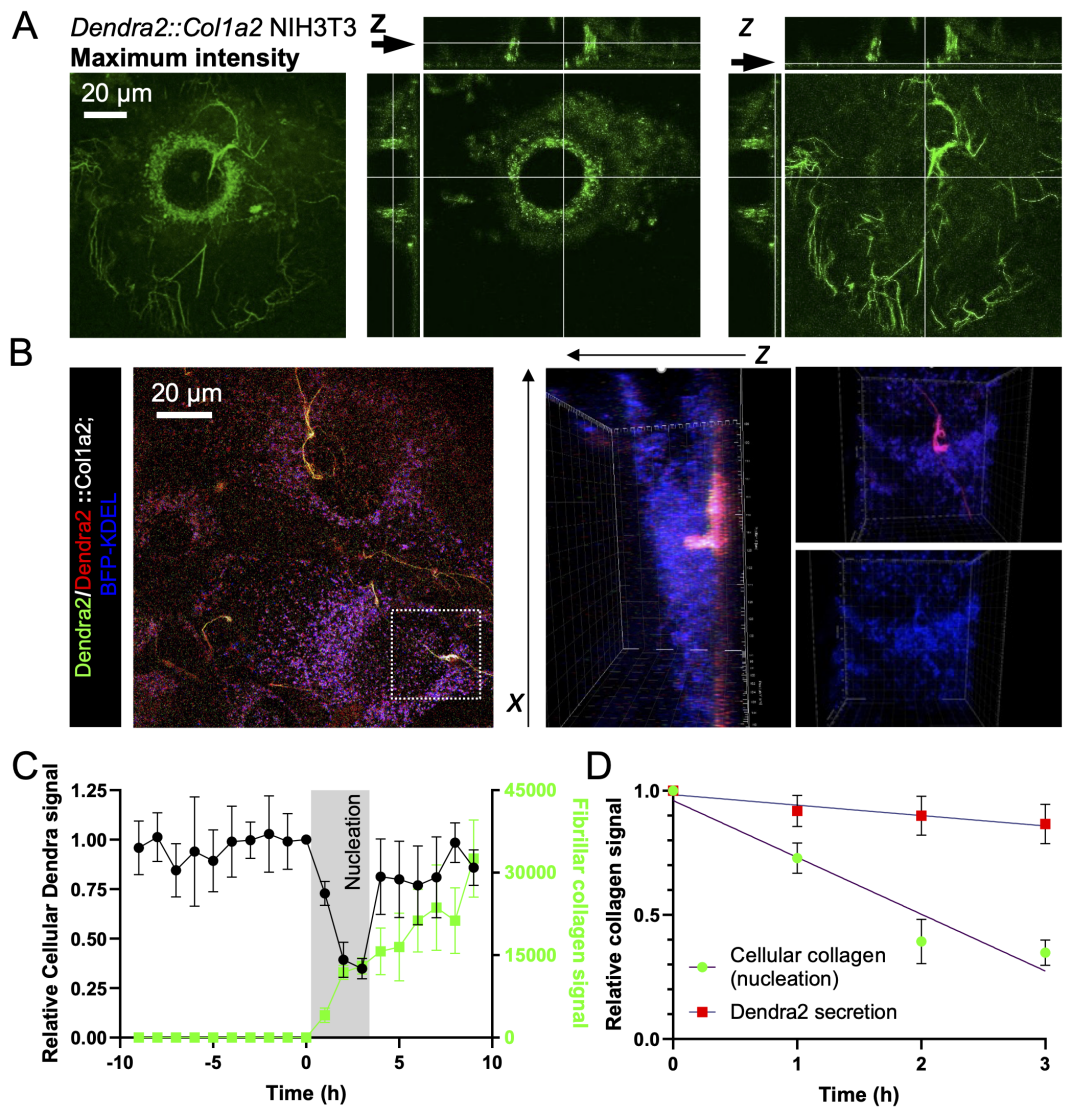
Collagen fibrils are formed between the basal surface of fibroblasts and the culture dish. **A**) Airyscan microscopy of Dendra2::Col1a2 NIH3T3 cells demonstrating that Dendra2 positive fibrils were formed within the boundary of the cell body. Images were captured after 48 h of culture. The maximum intensity projection of 51 different Z positions is shown on the left, the middle panel shows the localization of Dendra2::Col1a2 in a single Z plane within the cell body, and the right panel shows that Dendra2 fibrils are abundant on the basal surface of the cells, and a single Z plane is shown. The arrows indicate the Z-position of each image. Extended data 10 shows all 51 Z positions. **B**) Expression of BFP-KDEL in Dendra2::Col1a2 NIH3T3 cells demonstrates that fibrils are formed on the basal surface of the cells. A maximum intensity projection of 42 z-positions is shown on the left. The middle panel shows that photoswitched Dendra2 positive fibrils were beneath the cell body; however, loops in collagen fibrils were observed extending into the cell body, as shown in Extended data 11. Importantly, at positions where loops are engulfed by the cell, the endoplasmic reticulum (BFP-KDEL) aligns with the fibrils. **C**) Quantification of cellular Dendra2::Col1a2 signal intensity in the cell body prior to fibril formation onset The cellular Dendra2::Col1a2 signal drops dramatically during nucleation but subsequently recovers as fibrils elongate. Nucleation was set at the frame prior to the first detection of Dendra2 positive fibrils, which was designated as T=0, the cellular Dendra2 signal was set as 1, and the background pixel intensity was set as 0. Data from five cells that produced collagen fibrils are shown. Average data points are shown, n=5 individual cells, and error bars represent SD. **D**) The loss of cellular Dendra2 signal observed in C demonstrates the rapid release of cellular collagen at the onset of fibril formation, and the rate of loss was compared to the rate of loss of the photoswitched Dendra2 signal in the absence of fibril formation. Average data points are shown, n = 5 individual cells, and error bars represent SD.

**Figure 4.**
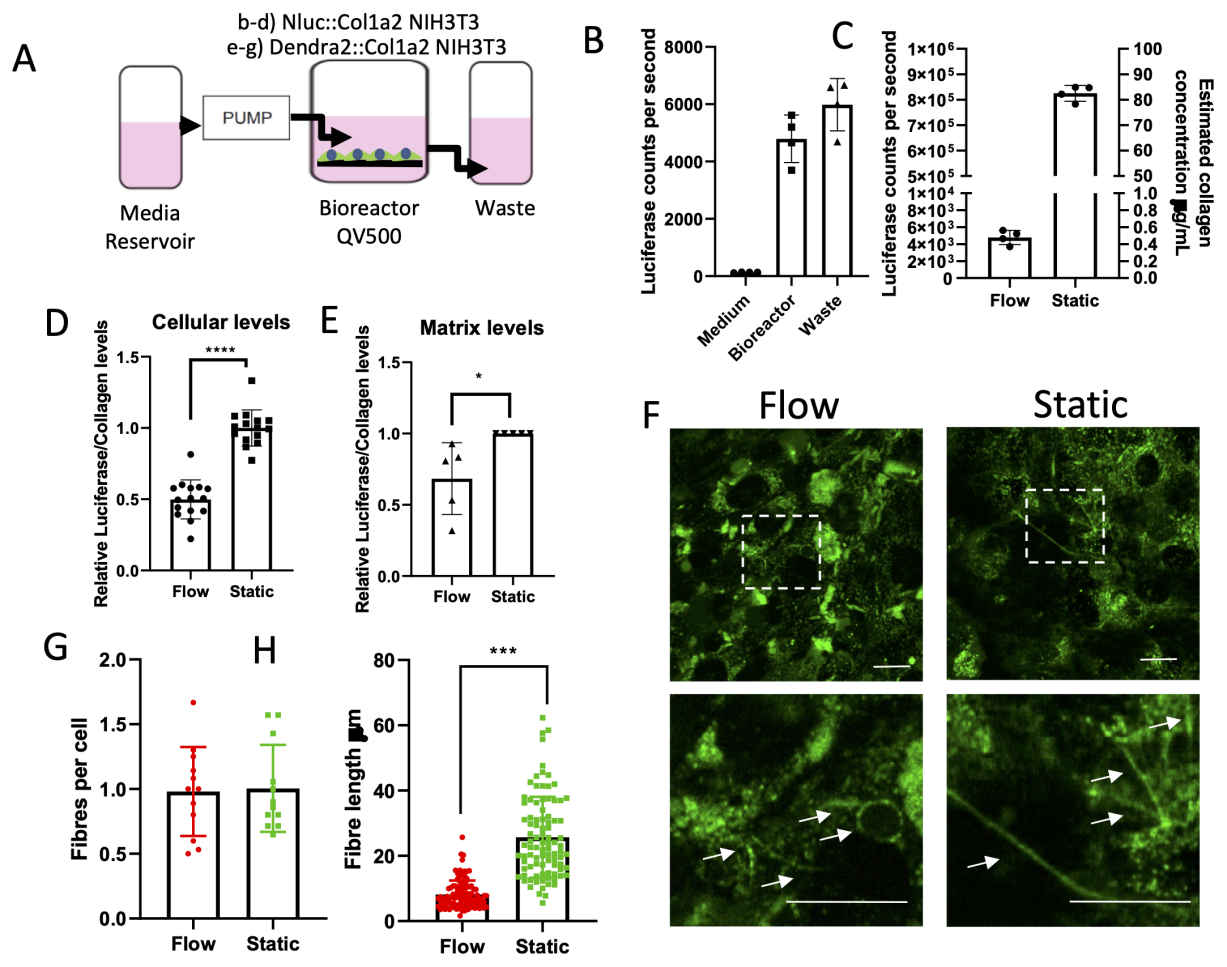
Rapidly secreted collagen contributes to fibril growth but not fibril nucleation. **A**) Schematic of the bioreactor used to study the effects of flow on fibril formation. **B**) To validate the flow system, Nluc::Col1a2 NIH3T3 cells were grown under the flow conditions. Nluc activity was assessed in the medium within the bioreactor housing the cells seeded on glass coverslips. Activity was also measured in the medium within the waste outflow (n=4 independent experiments; error bars show SD). **C**) Comparison of Nluc activity within the medium of Nluc::Col1a2 NIH3T3 cells grown under flow conditions or under static ‘normal’ culture conditions for 72 h. Comparison of Nluc activity in the medium was compared to recombinant Nluc protein to estimate the concentration of collagen within the medium as described previously ( Calverley *et al.*, 2020). **D**) After 72 h of culture, cells were removed from the bioreactor, coverslips were decellularized, and cellular Nluc activity was measured in triplicate (n=4 independent experiments, error bars show SD). **E**) After decellularization, the Nluc activity in the deposited matrix was also assessed. N=5 independent experiments; error bars show SD. **F**) In parallel experiments, collagen fibril deposition by Dendra2::Col1a2 NIH3T3 cells was assessed under flow or static conditions. Airyscan microscopy of live cells after 72 h of culture identified Dendra2 positive fibrils. Scale bar represents 20 µm) **G**) The number of deposited collagen fibrils was quantified per cell (n=3 fields per view) from 4 independent samples. **H**) The length of individual Dendra2 collagen fibrils was assessed using ImageJ image analysis software across all samples identified in **G**.

**Figure 5.**
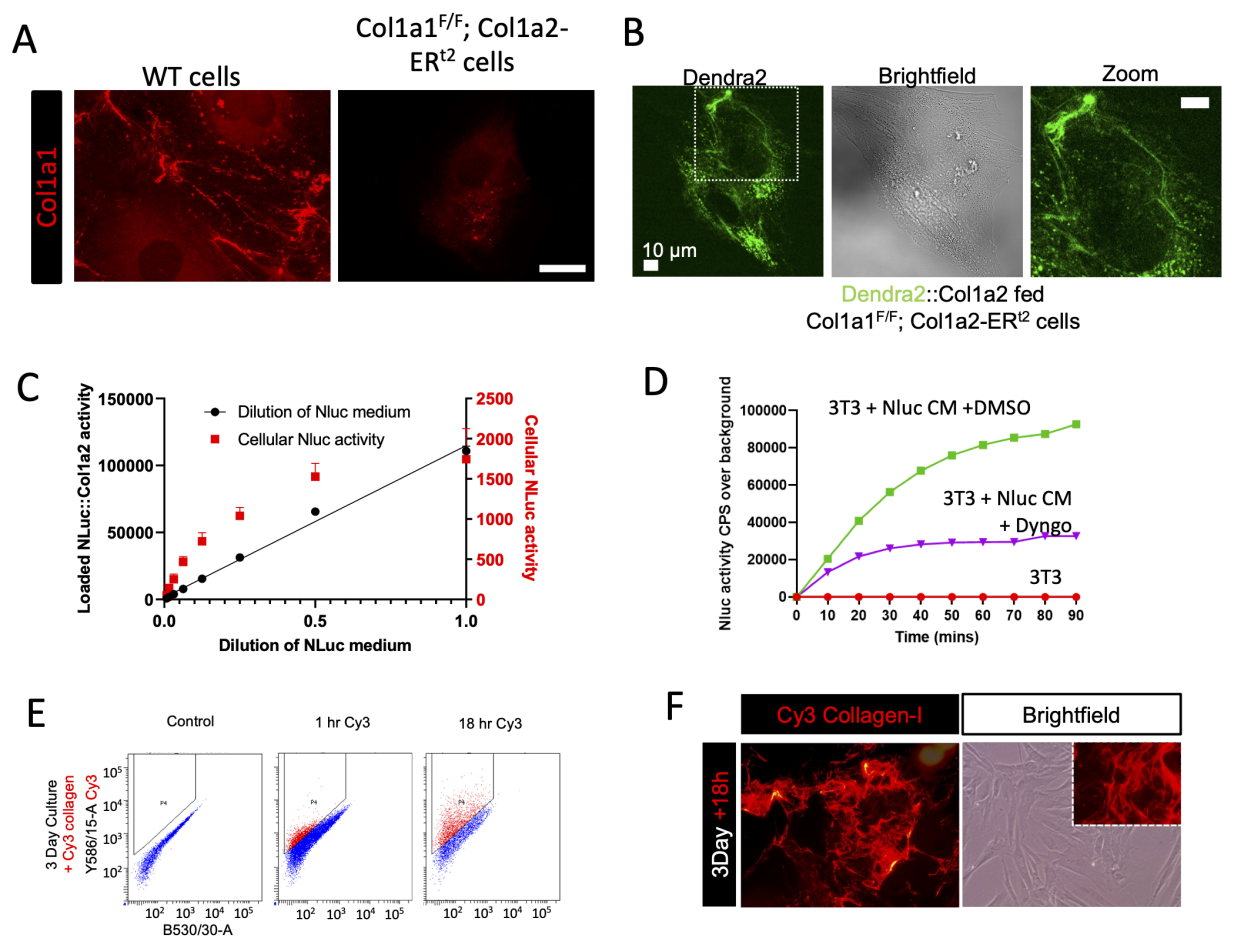
Endocytosed collagen contributes to fibril assembly. **A**) Deposition of type I collagen fibrils in cultures of tamoxifen-treated tendon fibroblasts from wild-type and Col1a1 ^F/F^; Col1a2-ER ^t2^ mice ( Li *et al.*, 2017; Yang *et al.*, 2013). Type I collagen was detected by immunofluorescence. **B**) Tamoxifen-treated fibroblasts from tendons of Col1a1 ^F/F^; Col1a2-ER ^t2^ mice were incubated with conditioned medium from Dendra2::Col1a2 NIH3T3 cells for 72 h. The medium was replaced daily. Dendra2 positive fibrils were only detected in the cells. Scale bar represents 10 µm. **C**) NIH3T3 fibroblasts were fed with conditioned medium from Nluc:Col1a2 NIH3T3 cells. The amount of Nluc activity taken up by tendon fibroblasts after 18 h was measured, demonstrating that cells were able to endocytose Nluc:Col1a2; however, there was a limit to the amount of Nluc activity that could be endocytosed. N=3 independent experiments; error bars show SD. **D**) NIH3T3 cells were seeded in 35 mm dishes and loaded with the live cell nanoluciferase substrate endurazine for 2 h, and then treated with DMSO or 10 µM Dyngo4a. Luminescence was then measured every 10 min after the addition of 1 mL of NLuc::Col1a2 conditioned medium cell-metabolized substrate. Representative traces are shown. N=3 independent experiments. **E**) Cy3 labelled rat tail collagen (10 µg/mL) was added to cultures of human foreskin fibroblasts for 1 or 18 h. Cells were detached and analyzed using flow cytometry to demonstrate time-dependent uptake by fibroblasts. Uptake was also observed in the SAOS2 cells and lung fibroblasts (Figure S3). **F**) Human foreskin fibroblasts incubated with 0.5 µg/mL Cy3 collagen for 18 h assembled labelled collagen into fibrillar structures.

**Figure 6.**
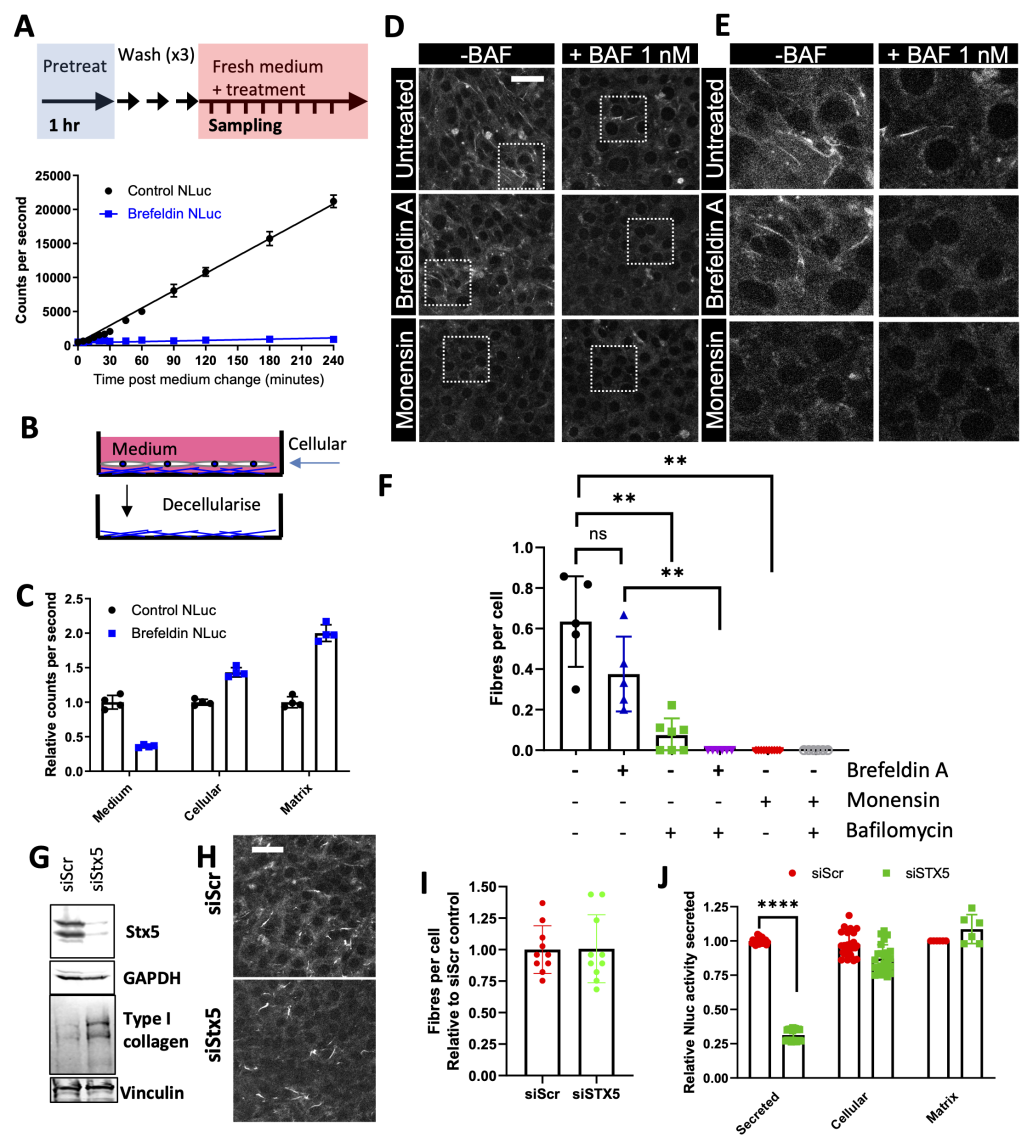
Non-classical PCI secretion feeds fibril assembly. **A**) Schematic for monitoring Nluc secretion from Nluc::Col1a2 NIH3T3 cells. Comparison of Nluc secretion rates in control cultures and cells treated with 100 nM brefeldin A revealed a robust reduction in Nluc secretion. **B**) Schematic to quantify the deposition of Nluc::Col1a2 into the extracellular matrix. **C**) Nluc::Col1a2 NIH3T3 cells were cultured with 100 nM brefeldin A for 72 h, and the Nluc activity levels in the conditioned medium, cellular fraction, and matrix were assessed. N=3 independent experiments and N=4 technical repeats from a representative experiment are shown. Error bars represent the standard deviation. **D**) Representative Airyscan confocal microscopy images of Dendra2::Col1a2 NIH3T3 cells cultured for 72 h in the presence of either 100 nM brefeldin A or 1 µM monensin, with or without the addition of the lysosome proton pump inhibitor 1 nM bafilomycin. Dendra2 signals highlight the deposited fibrils. The maximum intensity projections of the five z planes are shown. The scale bar represents 50 µm. Boxes are enlarged in E. **E**) Enlargements of images within the regions highlighted in D. **F**) Quantification of Dendra2 fibril numbers per cell, n=5 independent experiments for brefeldin and monensin treatment, with an additional n=2 independent experiments for treatments including bafilomycin. A minimum of 200 cells per condition per experiment were scored. Error bars represent SEM. ** represents p<0.01, Student’s T-test, unpaired. **G**) Western blot of syntaxin 5 (Stx5) knockdown with 100 pmol siRNA in NIH3T3 cells. N=3 independent experiments. **H**) Syntaxin 5 (Stx5) knockdown with 100 pmol siRNA in Dendra2::Col1a2 NIH3T3 cells was cultured for 72 h before live imaging of Dendra2 fibrils. The maximum intensity projections of the five z planes are shown. The scale bar represents 50 µm. **I**) Quantification of Dendra2 fibril numbers per cell in siRNA-treated Dendra2::Col1a2 NIH3T3 cells n=2 independent. Error bars represent SD. **J**) Syntaxin 5 (Stx5) knockdown with 100 pmol siRNA in Nluc::Col1a2 NIH3T3 cells was cultured for 72 h, and Nluc activity levels in the conditioned medium, cellular fraction, and matrix were assessed. N=3 independent experiments, each with N=4 technical repeats, are shown for conditioned medium and cellular fractions or n=2 technical repeats for matrix measurements. Error bars represent the standard deviation.

**Figure 7.**
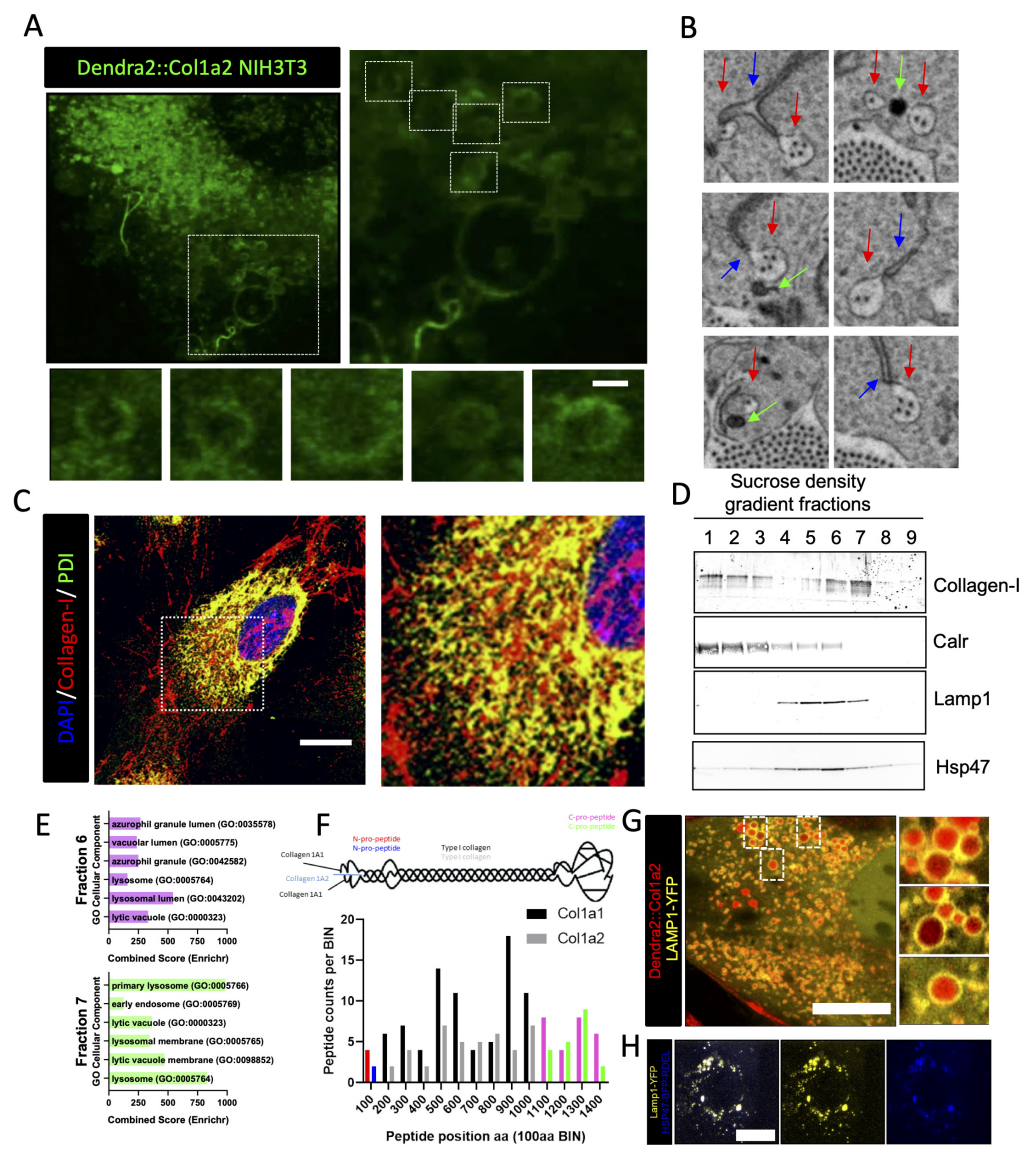
PCI localizes to LAMP1-positive compartments. **A**) Dendra2::Col1a2 NIH3T3 cells were imaged using Airyscan microscopy after 18 h of culture, 1000x magnification, and 2.5 × digital zoom; a maximum intensity projection is shown. Vesicles containing Dendra2 signals were observed at the sites of fibril assembly. The Dendra2 signal was observed at the periphery of the vesicles; the scale bar represents 1 µm. The area outlined by the box is enlarged in the right-hand panel. Small panels show looped structures containing Dendra2-positive collagen. **B**) Individual frames taken from Extended data 14 demonstrate that a single fibripositor (highlighted in the red box) is in contact numerous times by both the endoplasmic reticulum and electron-dense vesicles. The Golgi apparatus did not contact fibripositor (Extended data 15). **C**) Immunofluorescence imaging of type I collagen in mouse embryonic fibroblasts using the ER marker PDI. Scale bar represents 20 µm. **D**) Fractionation of NIH3T3 cells using a sucrose density gradient, type I collagen is co-resident with the ER protein calreticulin (Calr), but also with the lysosomal protein Lamp1 and the collagen chaperone Hp47. **E**) Proteomic analysis of lysosomal fractions 6 and 7 revealed significant enrichment of proteins identified by proteomic analysis of fractions 6 and 7 based on GO Cellular component terms. **F**) Col1a1 (Red and Magenta) and Col1a2 (Blue and Green)-derived procollagen peptides were present in fractions 6 and 7, suggesting that the newly synthesized collagen transitioned to these compartments. **G**) Dendra2::Col1a2 NIH3T3 cells were imaged by Airyscan microscopy 48 h after transfection with LAMP1-YFP. Images were recorded at 1000x magnification using a 2.5 × digital zoom. The maximum intensity projection of the 31 images is shown. LAMP1-YFP vesicles (green) containing photoswitched Dendra2 signals were observed. Three LAMP1-positive areas were also observed. **H**) Live super-resolution microscopy of NIH3T3 cells stably transduced with Hsp47-BFP-RDEL lentivirus and transfected with LAMP1-YFP. Scale bar, 20 µm.

Images for Figure 2A, Figure 3A, Figure 3B, Figure 4F, Figure 5B, Figure 6D, Figure 6E, Figure 6H, Figure 7A, Figure 7G, Figure 7H. Extended data 2B, 4B, 6A, 6B, Videos 2–5 were acquired with either a Fluar 20X 0.75NA objective at 1024 × 1024 resolution, or 100x using a Zeiss LSM880 equipped with Airyscan detector set to super-resolution mode. Green fluorescence was excited at 488 nm and collected using a 495–550 nm filter. The 32 phase images were recombined using the Airyscan processing tool in Zeiss Zen 2 software, and the image brightness and contrast were adjusted using BestFit.

Figure 5A, Figure 7C, Images in panels A and B were collected using a Leica TCS SP5 AOBS inverted confocal microscope with a [63x / 0.50 Plan Fluotar] objective. The confocal settings were as follows: pinhole [one airy unit] and scan speed [1000 Hz unidirectional]. Images were collected using [PMT] detectors with the following detection mirror settings; [FITC 494-530 nm; Cy5 640–690 nm] using the [488 nm (20%) and 633 nm (25%)] laser lines, respectively. To eliminate crosstalk between the channels, images were sequentially collected.

### Electron microscopy

Transmission electron microscopy: Dendra2::Col1a2 edited MC3T3 cells were grown for 7 days in the presence of 200 µg/mL l-ascorbic acid, replenishing medium, and ascorbic acid every 2 days. Following fixation in 2.5% glutaraldehyde/100 mM phosphate buffer (pH 7.2) for 30 min at RT, the matrix was scraped from the culture dish. After 2 h at 4 °C, samples were washed with 100 mM phosphate buffer (pH 7.2). After immersion in 2% osmium/1.5% potassium ferrocyanide in cacodylate buffer (pH 7.2) for 1 h at RT, samples were washed in ddH2O and fixed in 1% tannic acid/0.1 M cacodylate buffer (pH 7.2) for 2 h at 4 °C. Samples were then thoroughly washed in ddH2O and incubated in 2% osmium tetroxide/ddH2O for 40 min at RT before washing again in ddH2O at RT. This was followed by a final overnight incubation step at 4 °C in 1% uranyl acetate (aqueous). Samples were then washed before infiltration with a series of propylene oxide and TAAB 812 resin kit mix, with increasing resin concentration (2 h in 30% resin, 2 h in 50% resin, 2 h in 75% resin, and 3 × 1 h in 100% resin). samples were then embedded in capsules and cured at 60 °C for 12 h. Sections (80 nm thick) were cut from the sample blocks and examined using a Tecnai 12 BioTwin electron microscope.

Serial block face SEM: Tendons were prepared as previously described ( Starborg *et al.*, 2013). In brief, tendons were fixed in 1% osmium and 1.5% potassium ferrocyanide in 0.1 M sodium cacodylate buffer for 1 h, washed with ddH _2_O water, incubated with 1% tannic acid in 0.1 M cacodylate buffer for 1 h, washed, and then incubated with 1% osmium tetroxide in water for 30 min. Samples were then washed with ddH _2_O, stained with 1% uranyl acetate in water for 1 h, dehydrated in acetone, and embedded in resin.

ImmunoEM: Post-embedded labelling was used to detect type I collagen using a rabbit anti–chicken collagen-I antibody (Biodesign International) at a dilution of 1:500, followed by a gold-conjugated goat anti–rabbit antibody (British Biocell International) at a dilution of 1:200. All sections were subsequently stained with uranyl acetate and examined under a Tecnai 12 BioTwin electron microscope.

### Atomic force microscopy

Atomic force microscopy was performed using a JPK NanoWizard IV (Bruker Nano Inc., Karlsruhle, Germany, JPK Instruments AG, Berlin, Germany) mounted on a Zeiss AX10 inverted light microscope (Carl Zeiss Microscopy GmbH, Jena, Germany) using JPK NanoWizard Control software (V 6.1.65). Images were captured using NuSense Scout 350R cantilevers (NuNano, Bristol, UK) with a nominal spring constant, frequency, and tip radius of 42 N/m, 350kHz and < 10 nm, respectively. Height data were processed using JPK Data Processing software (V 6.1.65), and was 1 ^st^ order flattened prior to analysis.

### Nanoluciferase activity assay

Conditioned medium collected after washing edited cells twice with PBS, was placed in white walled 96 well plates (Nunc MicroWell 96-Well, Nunclon Delta-Treated, Flat-Bottom Microplate, Thermo Fisher Scientific, Paisley, UK# 136101) To assay Nluc activity 0.5 µL of coelenterazine (final concentration 3 µM) was added immediately prior to measurement. Light production was measured using filter cubes #114 and #3 on the Synergy Neo2 Multi-Mode Reader (Biotek), readings for each well were integrated over 200 ms with 4 replicate measurements per well (Gain 135 and read height 6 mm). For assessment of cellular and matrix-derived Nluc activity, cultures were decellularized by the addition of extraction buffer (20 mM NH _4_OH, 0.5% Triton X-100 in PBS) for 2 min at 37 °C until no intact cells were visible under a light microscope, the cellular fraction was removed, the matrix was washed with PBS, scraped into 1 mL PBS, and pelleted by centrifugation at 12,000 × *g* for 5 min. The matrix was resuspended in 100 µL of extraction buffer before assessing the Nluc activity.

### Hydroxyproline assay

Decellularized cultures were scraped and pelleted into 1.5 mL tubes before freezing at -20 °C for the hydroxyproline quantitation. The hydroxyproline content was measured using previously described methods ( Reddy & Enwemeka, 1996). Briefly, 100 µL 6M HCl was added to the pellet and incubated at 100 °C overnight. The samples were cooled to room temperature and spun at 12,000 *xg* for 3 min to remove the residual charcoal. Each sample (50 μL) was mixed with chloramine-T (450 μL) and incubated at room temperature for 25 min. Ehrlich’s reagent (500 μL) was added to each sample and incubated at 65 °C for 10 min. All samples were compared to hydroxyproline standards that were treated identically. The absorbance of 100 μL was measured in a 96-well plate, and the absorbance at 558 nm was read on an H1 plate reader (Biotek).

### Lysosomal fractionation

Cells (30 million) were grown in 150 mm tissue culture dishes, trypsinized, and pelleted at 1000 *xg* for 5 min. Lysosomal fractions were prepared using a Lysosome Isolation Kit (Sigma Aldrich, LYSISO1) according to the manufacturer’s instructions. Nine fractions were collected following ultracentrifugation.

### Proteomic sample preparation

Cell pellets were resuspended in 30 µL of SL-DOC (1.1% sodium laurate and 0.3% sodium deoxycholate in 25 mM ammonium bicarbonate supplemented with protease and phosphatase inhibitor cocktails). Six 1.6 mm steel beads were added, and the samples were homogenized in a Bullet Blender Tissue Homogenizer. A BCA was performed to quantify the amount of protein in each sample. Each sample (50 µg) was made up to 5% SDS, and then reduced and alkylated with DTT and Iodoacetamide (IAA), respectively. For the lysosome fractions, the samples were processed without further extraction. Samples were acidified using H _3_PO _4 _and S-trap binding buffer (90% methanol in 100 mM TEAB, pH 7.1) was added. Samples were loaded onto S-Trap columns (ProtiFi) and washed four times with S-trap binding buffer 4 times. Proteins were digested with 0.8 µg/µL trypsin solution (Proteomics grade trypsin, Promega). The peptides were then eluted in 65 µL of digestion buffer (50 mM TEAB, pH 8.5), 65 µL of 0.1% formic acid (in water), and 30 µL of 0.1% formic acid and 30% acetonitrile (ACN) (in water). Samples were then desalted using Oligo R3 resin beads in a 96-well, 0.2 µm PVDF filter plate (Corning). The beads were washed, and then the samples were added and washed twice with 0.1% formic acid. The samples were then eluted in 0.1% formic acid in 30% ACN and lyophilized using a speed-vac (Heto Cooling System).

### Mass spectrometry

The dried peptides were resuspended in 10 µL of 0.1% formic acid in 5% ACN. Samples were analyzed using an UltiMate ® 3000 Rapid Separation LC system (RSLC, Dionex Corporation) coupled to an Orbitrap Elite for quality control and then a Q Exactive HF Mass Spectrometer (Thermo Fisher). For both, mobile phase A was 0.1% formic acid in water and B was 0.1% formic acid in ACN. The Orbitrap used a 75 mm x 250 um inner diameter 1.7 µM CSH C18 analytical column (Waters) with a gradient from 92% A and 8% B to 33% B in 10 minutes at a rate of 300 nL/min. Q Exactive used a gradient of 95% A and 5% B to 18% B at 58 min, 27% at 72 min, and 60% at 74 min with the same flow rate and a 75 mm × 250 µm inner diameter CSH C18 analytical column (Waters). Peptides were automatically selected by DDA for fragmentation, and data were acquired for 90 min in the positive mode.

### SILAC labelling

Mouse tendon fibroblasts were pulsed with 100 mg/L C ^13^ (“Heavy”) lysine for 48 h, washed, trypsinized, lysed, and processed for mass spectrometry (see below). Mass spectrometry result files were exported into Proteome Discoverer for identification and quantification using the SILAC 1plex (Lys6) method. All searches included a fixed modification of carbamidomethylation of cysteine residues resulting from IAA treatment to prevent cysteine bonding. The variable modifications included in the search were oxidized methionine (monoisotopic mass change, +15.955 Da) and the phosphorylation of threonine, serine, and tyrosine (79.966 Da). A maximum of two missed cleavages per peptide were allowed. The minimum precursor mass was set to 350 Da with a maximum of 5000 Da. Precursor mass tolerance was set to 10 ppm, fragment mass tolerance was 0.02 Da and minimum peptide length was 6. Peptides were searched against the SwissProt database using Sequest HT, with a maximum false discovery rate of 1%.

Half-lives were calculated from heavy to light ratios (HL) using the following equations ( Schwanhausser *et al.*, 2009):

$$k=\frac{\ln\left(HL+1\right)}{48}-\frac{\ln\left(2\right)}{72}$$

$$t_{1\text{/}2}=\frac{\ln\left(2\right)}{k}$$

where 48 h represents the SILAC pulse time, 72 h is the calculated cell doubling rate, and k is the rate constant of protein decay, which is used to calculate the half-life in the second equation.

This calculation assumes that no new light protein is produced, and that the amount of light protein decays exponentially over time. The total amount of protein was assumed to be double per complete cell cycle.

Proteins were only selected for half-life calculation if at least three peptides were detected, where at least one peptide was heavy, across two repeats.

## Results

### Engineering of tagged procollagen-I in NIH3T3 cells

In this study, we employed CRISPR-Cas9 technology to engineer photoswitchable Dendra2 or nanoluciferase (Nluc) downstream of the signal peptide sequence of proa2(I) in NIH3T3 cells (shown schematically in Figure 1A), resulting in the expression of the tagged procollagen I (PCI) protein. Our quantification analysis revealed that fibroblasts secrete approximately 100,000 procollagen molecules per hour, which is equivalent to their entire procollagen content in approximately 2.5 hours (Extended data 1). Detection of Nluc-tagged PCI in the medium within 5 min of changing the culture medium confirmed rapid secretion rates (Extended data 1B), which is consistent with previous findings ( Canty *et al.*, 2004; Mirigian *et al.*, 2014), highlighting the significant synthetic capacity of fibroblasts. 

### Tracking the fate of procollagen-I molecules

To track the fate of procollagen molecules, we used Dendra2 to tag PCI at the N-terminus (Extended data 1C-F). The insertion of Dendra2 maintained allele responsiveness to pro-fibrotic stimuli (Extended data 1G) and did not alter secretion rates, as assessed by the insertion of a HiBit tag into exon 6 using CRISPR-Cas9 ( Figure 1B). Leveraging the photoswitchable properties of Dendra2, we switched’ the PCI’ ( Figure 1C) and followed the fate of existing PCI (which had been ‘switched’ to red) and the fate of newly-synthesized PCI (which was ‘green’). During 48 h, pre-existing Dendra2-PCI (red) was secreted, whereas some of the newly synthesized Dendra2-PCI (green channel) remained in the cell ( Figure 1D). Photoswitched Dendra2-PCI exhibited a half-life of approximately 24 h ( Figure 1E). These results indicate that not all PCI are rapidly secreted. Time-lapse analysis revealed that newly synthesized PCI was initially located in the puncta before becoming diffuse throughout the cell between 24 and 48 h (Extended data 2). At higher magnifications, newly synthesized Dendra2-PCI transitioned through the Golgi apparatus, while a large cellular pool of pre-existing Dendra2-PCI (yellow) remained within the cell for over 9 h ( Figure 1F).

### An intracellular pool of PCI confirmed by SILAC

To assess the residence time of PCI in cells, we utilized Stable Isotope Labelling by Amino Acids in Cell Culture (SILAC) labelling. This approach enables measurement of protein turnover rates and secretion dynamics. Unedited NIH3T3 fibroblasts were cultured for 48 h with C13-lysine, followed by analysis of intracellular proteins using liquid chromatography coupled tandem mass spectrometry (LC-MS/MS) ( Figure 1G). The analysis revealed the presence of 'heavy' and 'light' peptides for 1380 proteins. Notably, both 'heavy' and 'light' Col1a1-encoding peptides were detected after 48 hours of labelling, with 39–43% of the peptides being 'light' ( Figure 1H), which supported the existence a longer-lived pool of intracellular PCI. The mass spectrometry proteomics data were deposited to the ProteomeXchange Consortium via the PRIDE ( Perez-Riverol *et al.*, 2022) partner repository with the dataset identifiers PXD036794 and 10.6019/PXD036794.

The ratio of 'light' to 'heavy' peptides facilitated the determination of protein half-lives ( Figure 1I). The presence of both 'heavy' and 'light' peptides derived from proa1(I) suggested the existence of a stable PCI pool (resulting in 'light' peptides) and a PCI pool synthesized during the 48-hour C13-lysine labelling ('heavy' peptides). The ratio of light/heavy peptides allowed for the calculation of the half-life of proa1(I), which was found to be 69.8 hours.

Comparisons with other proteins revealed a wide range of half-lives, with Hnrnpa1, involved in pre-mRNA packaging, exhibiting one of the longest half-lives (~123 h), whereas Lig1, a DNA ligase, had a half-life of ~34 h. This identification of a long-lived pool of PCI in unedited cells corroborates the microscopic data obtained using Dendra2-edited cells. However, the discrepancy in half-life estimates between imaging and SILAC labelling suggests that fibroblasts may employ additional mechanisms to re-uptake secreted proteins (which we explored in experiments, described below), thereby lengthening the cellular residency of 'light' peptides.

### Collagen fibrils assemble at the plasma membrane

Dendra2-labelled collagen fibrils were not observed within the first 24 h of imaging; however, with extended culture, Dendra2-positive fibrils were visible approximately 36 h after cell plating ( Figure 2A). Multiple fibrils appeared simultaneously and grew in length over the next 12 hours (Extended data 3). This time lag in fibril formation aligns with previous findings using fixed samples ( Chang *et al.*, 2020; Kubow *et al.*, 2015). Correlative Airyscan microscopy and atomic force microscopy (AFM) confirmed that Dendra2-positive fibrils exhibited a *D*-period of 65 ± 7.4 nm, which was within the expected range for collagen fibrils ( Ushiki, 2002) ( Figure 2B, C). These fibrils were confirmed to be type I collagen by immunofluorescence detection (Extended data 4). Similar to the effect of Dendra2 tagging on type I secretion, the location of the fluorescent protein did not impede fibril assembly. A key observation was that Dendra2-positive collagen fibrils were always associated with cells ( Figure 3A, Extended data 5); fibrils did not form independently of cells, as might have been predicted from *in vitro* fibril assembly studies. Optical sectioning of the cultures and visualization of the cell body using an ER-targeted blue fluorescent protein (KDEL-BFP) revealed that Dendra2-positive fibrils were deposited on the basal cell surface, near but distinct from the BFP signal ( Figure 3B, Extended data 6).

Insights into how the cellular pool of collagen contributes to the assembly process were also captured in these experiments: Cellular Dendra2-PCI levels decreased rapidly during fibril nucleation, at a rate five times greater than that observed when assessing secretion ( Figure 3B, C). Further examination of KDEL-BFP-transfected cells demonstrated close proximity of the ER with the extracellular collagen fibril, with the ER arranged aligned to the forming collagen fibrils, suggesting that these sites may contribute to rapid secretion. We aso observed looped fibrillar structures engulfed by the cell body, which were often observed to be pulled by migrating cells. These images reinforce the conclusion that collagen fibril assembly is under strict cellular control and involves the plasma membrane in close association with secretion machinery.

### Fibril nucleation is not dependent on classical collagen secretion

To generate further understanding, we employed a bioreactor to apply a constant flow of culture medium over the apical surfaces of CRISPR edited NIH3T3 cells and thereby maintaining a low extracellular concentration of collagen ( Figure 4A). Applying a constant flow rate (0.05 mL/minute) throughout culturing significantly reduced extracellular media collagen levels by 99.5% when compared to static conditions ( Figure 4B–C). The decellularization of the cell monolayer maintained under constant flow showed reduced cellular levels of Nluc ( Figure 4D) and reduced incorporation of Nluc into the matrix ( Figure 4E). Fibril assembly, assessed by microscopy, of Dendra2::Col1a2 NIH3T3 cells showed that the number of collagen fibrils per cell was not significantly affected by the flow ( Figure 4F, G). The reduced cellular levels of Nluc in the face of unchanged collagen fibril numbers were explained when measurements of Dendra2 positive fibrils showed significantly shorter fibrils when cultured under flow conditions. Together, these results demonstrate that secreted collagen contributes to fibril growth but that nucleation is controlled and driven by cells.

Having established that fibril nucleation is under cellular control, we next sought to understand how the secreted collagen contributes to fibril growth. This was assessed in two ways: i) elimination of collagen nucleation sites, and ii) addition of exogenous collagen. First, to eliminate fibril nucleation sites, we crossed Col1a1fl/fl mice ( Li *et al.*, 2017; Yang *et al.*, 2013) with Col1a2-CreERT(2) mice ( Li *et al.*, 2017; Yang *et al.*, 2013) and isolated tendon fibroblasts. The primary fibroblasts were then incubated with tamoxifen to stop endogenous full-length collagen production. Immunofluorescence showed no type I collagen fibrils after tamoxifen treatment ( Figure 5A). When these cells were treated with conditioned medium from Dendra2:Col1a2-edited NIH3T3 cells, tagged collagen was observed intracellularly and assembled into Dendra2-PCI into fibrils ( Figure 5B). This observation suggests that Dendra2:Col1a1 collagen was endocytosed by the cells and repurposed into fibrils. The endocytosis of collagen was confirmed by the addition of Nluc-PCI-conditioned media to NIH3T3 cells ( Figure 5C), which was found to be an active process, unlike the uptake of the untagged Nluc enzyme (Extended data 7). Uptake of Nluc-PCI was dynamin-dependent, as evidenced by Dyngo treatment ( Figure 5D). The observation that endocytosed collagen contributes to fibril growth was confirmed by the addition of Cy3-labelled rat tail collagen to NIH3T3 cultures; not only did the cells uptake Cy3-labelled collagen as assessed by flow cytometry ( Figure 5E), they also assembled this into the extracellular matrix ( Figure 5F). The uptake was also confirmed in human lung fibroblasts and SAOS2 cells (Extended data 7). These data support our earlier findings that extracellular collagen is endocytosed, which likely contributes to the longer than anticipated half-life of cellular collagen identified by SILAC labelling. Furthermore, these data demonstrated that the uptake of collagen from the extracellular space contributes to fibril assembly. This process functions independently from the collagen synthesis pathway and provides new understanding of how the extracellular matrix is assembled.

### Collagen fibril formation blocked in the presence of monensin but not brefeldin A

To further examine how cells assemble collagen fibrils, we utilized Brefeldin A inhibit ER-Golgi transport ( Colanzi *et al.*, 2013). Brefeldin A inhibited the secretion of Nluc-PCI into the culture medium ( Figure 6A) but did not result in a reduction in collagen incorporation into the extracellular matrix ( Figure 6B, C). This finding was confirmed when cultures of Dendra2:Col1a2 cells were grown in the presence of brefeldin A and indicated that pathways beyond the classical ER-Golgi secretory pathway might be involved in collagen fibril assembly. Next, we used monensin to inhibit both ER-Golgi transport and transport to the lysosomes ( Stenseth & Thyberg, 1989). Monensin blocked fibril formation ( Figure 6D–F), suggesting that transport of collagen to the lysosome may be required for fibril formation. To explore whether the lysosome compartment plays a role in fibril assembly, the inhibitors of lysosome function, bafilomycin A1 and chloroquine (reviewed by ( Fedele & Proud, 2020)) were applied to unedited fibroblasts. At doses that did not alter cellular proliferation, both bafilomycin A1 and chloroquine significantly inhibited fibril formation and fibril length, as assessed by immunofluorescence and total collagen deposition assessed by hydroxyproline quantification (Extended data 8). When the combination of Bafilomycin and Brefeldin A applied, fibril formation was completely blocked, suggesting that ER-Golgi and lysosomal trafficking are required for collagen fibril formation ( Figure 6F). This was also supported by more targeted interruption of only the ER-Golgi traffic, where knockdown of syntaxin 5 (STX5) disrupted only procollagen secretion but had no impact on collagen incorporation into the matrix ( Figure 6G–J, Extended data 9).

### Dendra2-PCI is transported in Lamp1-positive carriers

The possible involvement of lysosomal or lysosomal-like compartments in collagen fibril assembly has motivated additional experiments. We used confocal microscopy with Airyscan detection to live image Dendra2:Col1a2 cells to visualize pre-fibril-forming events. Dendra2-PCI was conspicuously localized in a constellation of punctate compartments ( Figure 7A). These compartments were closely associated with collagen fibrils, which also formed loop structures ( Figure 7A, insert). Such a close association of collagen fibril-containing fibripositors with darkly stained compartments (presumed to be lysosomal compartments) is frequently observed in electron microscopy images of embryonic tendons ( Figure 7B) ( Canty *et al.*, 2004). In tendon fibroblasts cultured for 72 h, intracellular PCI localized largely to the ER, as indicated by co-localization with the ER-resident enzyme, protein disulfide isomerase (PDI) ( Figure 7C). There were also regions where the two proteins did not colocalise. In one set of experiments to determine the identity of these compartments, we performed cellular fractionation, followed by western blotting and LC-MS/MS protein identification. As expected, PCI was identified in fractions that contained ER-resident proteins (e.g., calreticulin, CALR) ( Figure 7D). PCI was also found in fractions that did not contain CALR (see fractions 5–7 in Figure 7D). Some PCI-positive fractions contain lysosome-associated membrane protein (LAMP)-1, which also contains Hsp47, which binds to triple helical collagen ( Ishikawa & Bachinger, 2013). These PCI-positive/CALR-negative fractions were analyzed using LC-MS/MS (Extended data 10). Pathway analysis of the proteins identified ‘endocytosis’ and lysosomes as enriched pathways ( Figure 7E). Peptides originating from LAMP1 and LAMP2, as well as core components of the retromer complex (Vps35, Vps26, and Vps29) and the sorting nexins SNX1 and SNX6 were present in these PCI-containing fractions (Extended Data 10). The retromer is responsible for the recycling of transmembrane proteins to the cell surface or trans-Golgi network (TGN) and prevents their degradation by the lysosomal system ( Bonifacino & Hurley, 2008). Within these fractions, peptides for both proa1(I) and proa2(I) were identified, including peptides from N- and C-propeptides. Further analysis of the proteins within these fractions identified 35 known collagen-interacting proteins ( Doan *et al.*, 2019). These included Hsp47 and PPIB, which catalyze peptidyl proline isomerization and the rate-limiting step in the folding of the collagen triple helix. Similarly, Colgalt1, which transfers β-galactose to hydroxylysine residues on collagen, was also present. The collagen crosslinking enzymes, PLOD1-3, were also readily detected (Extended data 10). Taken together, the presence of these proteins suggests that triple-helical PCI molecules and the procollagen folding machinery are present at the sites of collagen fibril formation.

The presence of PCI and LAMP1 in fractions 6 and 7 in the sedimentation studies prompted us to investigate whether these molecules could be colocalized using imaging approaches. Therefore, we expressed LAMP1-YFP in the Dendra2:Col1a2 cells. Dendra2-PCI was readily detected in LAMP1 positive compartments ( Figure 7 G). LAMP1 was also located at sites close to the collagen fibrils (Extended data 11). Interestingly, Hsp47-BFP colocalized with LAMP1-YFP ( Figure 7H). Colocalization of collagen-I with LAMP1 was also confirmed in both mouse and human fibroblasts (Extended data 11).

### Disrupted lysosome function results in defective deposition of type I collagen to the matrix

**Figure 8.**
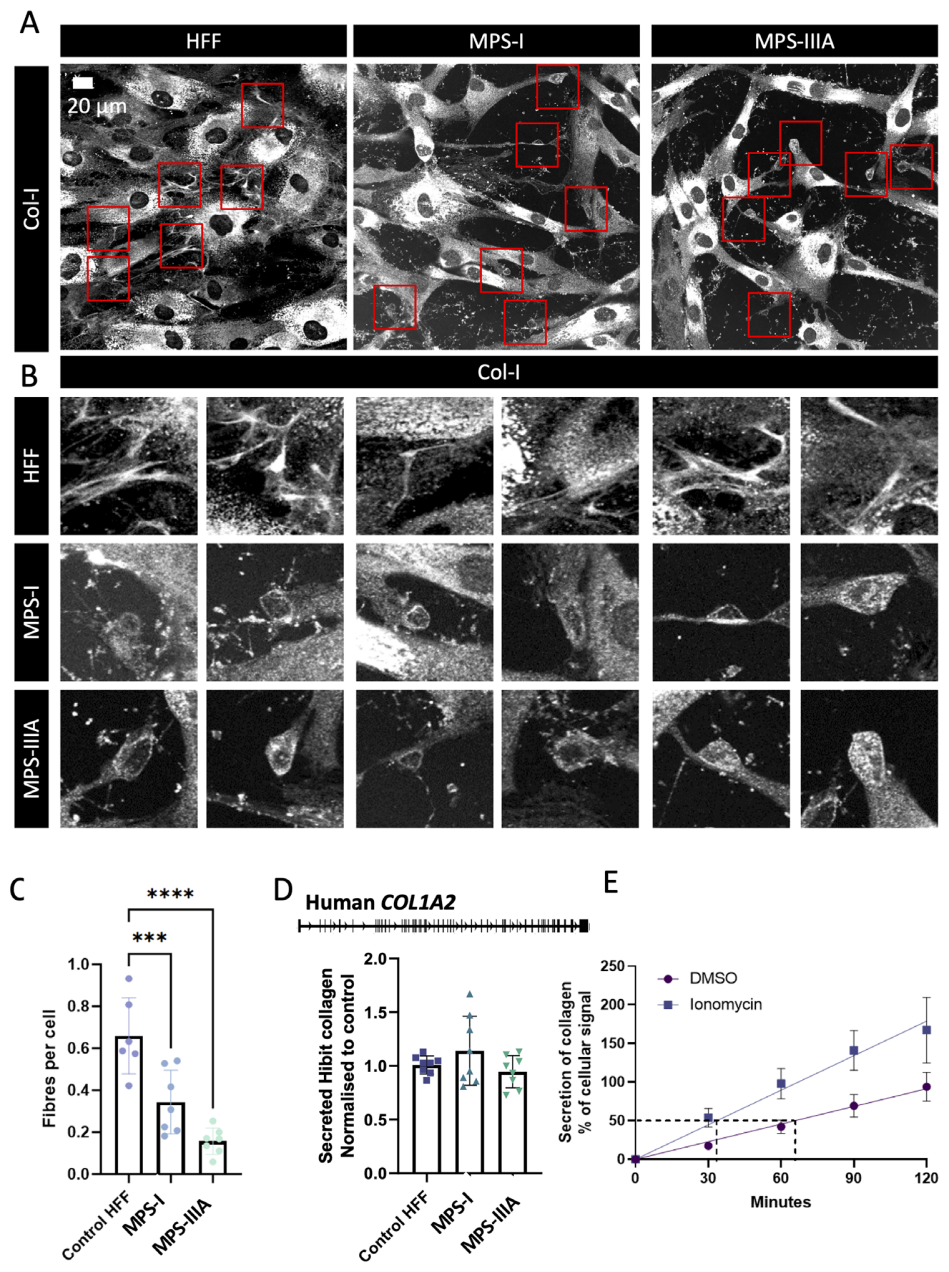
Disruption of type-I collagen fibril assembly in fibroblasts from patients with lysosome storage disorders. **A**) Immunofluorescence detection of type-I collagen in human skin fibroblasts obtained from individuals with lysosomal storage disorders, mucopolysaccharidosis type 1, and type 3a (MPSI and MPSIIIA). Scale bars, 20 µm. **B**) Regions highlighted in A show failed collagen assembly sites in MPS patient derived fibroblasts. **C**) Quantification of deposited collagen fibrils in control and MPS patient derived fibroblasts **D**) CRISPR-Cas9 mediated knock-in of the split nanoluciferase tag, Hibit, into the Exon 1 of Col1a2 in control HFF and MPS patient-derived fibroblasts. Secretion rates of Hibit-tagged collagen were normalized to cellular Hibit levels, indicating that there was no difference in the ability to secrete type I collagen. **E**) Ionomycin triggered the release of Nluc::Col1a2 from NIH3T3 cells. N=9 independent experiments; error bars represent SEM.

To further explore the possible role of lysosomal compartments in the deposition of type I collagen, we used dermal fibroblasts derived from patients with lysosomal storage disorders. Fibroblasts were isolated from patients with mucopolysaccharidosis (MPS) type I and type IIIA (MPS-I, W402X mutation in IDUA and MPSIIIA, R245H, and c1284del11 mutations) mutations, and lysosomal enzyme activity defects were confirmed in isolated fibroblasts ( **Extended data 12**). These fibroblasts were allowed to assemble into a matrix over 3 days. The detection of collagen fibrils by immunostaining with an anti-collagen I antibody revealed reduced deposition of collagen fibrils in these cultures. Failed fibril nucleation events were observed in both MPSI and MPSI IIA patient-derived fibroblasts ( Figure 8A–C, red boxes), implicating the lysosome as an essential compartment in the formation of collagen fibrils. Secretion was assessed using CRISPR-Cas9 mediated Hibit tagging. This approach revealed that PCI secretion was unaffected in these fibroblasts ( Figure 8D). The failed fibril formation events were reminiscent of the early fibril nucleation sites observed in the Dendra2-PCI experiments, as shown in Figure 2. In the final experiment, we showed that ionomycin (a lysosome inhibitor) led to increased intracellular PCI compared to untreated cultures ( Figure 8E). Taken together, this final set of experiments points to a conclusion in which the lysosome or lysosomal-like compartments act as a store of PCI in readiness for fibril formation. When this store is perturbed, either by gene mutations in the case of lysosomal storage disorders or by pharmaceutical intervention, fibril formation can be attenuated.

## Discussion

In this study, we established that cells control collagen fibril assembly by tethering one end of the fibril to the plasma membrane. We established a fundamental distinction between collagen molecule synthesis and fibril formation using a combination of gene editing and live light microscopy to label collagen. We showed that collagen *molecule* secretion occurs via the conventional ER-Golgi secretory pathway at a rate of 100,000 PCI molecules/h, but that collagen *fibril* assembly occurs at the plasma membrane every 24 h via an intracellular pool. We provide evidence that collagen molecules can be captured from the extracellular space and repurposed into fibrils via a route characterized by endosomal and lysosomal marker proteins. These findings help resolve the long-standing puzzle of how cells initiate a new collagen fibril and elongate one that has already been started. Our study highlights a critical distinction between collagen *molecule* secretion and *fibril* assembly.

The use of CRISPR-Cas9 to introduce photoswitchable Dendra2 into PCI enabled us to distinguish newly synthesized PCI (green) from pre-existing PCI (red after photoswitching) within the cell. Using this approach, we showed that PCI is stored within the cell in a collection of vesicles with t½ of 89 h. Rhythmically, every 24 h, the majority of PCI in the pool is directed to sites of fibril assembly at the plasma membrane. We considered that some of the features observed could be due to photobleaching. However, there was no bleed-through from the green channel into the red channel as the two were imaged on separate tracks on the confocal microscope with 488 and 561 excitations, respectively. Indeed, if there was any bleed-through in the time-lapse images of photoswitched Dendra2-PCI, this would be apparent in Figure 1. Sufficiently low levels of light were used to excite Dendra2 (at either wavelength) to minimize photobleaching. If there were any significant photobleaching, the free radicals produced would have precluded cell survival over the 48 h of the experiment, and both red and green signals from Dendra2 would have been lost. The photoswitching of Dendra2 was not 100% efficient at the intensities used, but sufficient photoswitched Dendra2 was visible to allow estimation of the half-life due to turnover and secretion. The fact that the Dendra2 signal diminished five times faster during fibril nucleation (e.g., Figure 3) supports the interpretation that the loss of the photoswitched Dendra2 signal from the cells was predominately due to secretion rather than any significant contribution from photobleaching and protein degradation.

The spatial distribution of vesicles comprising the PCI pool does not conform to our current understanding of the ER or Golgi apparatus. Indeed, fibril formation (which draws PCI from the pool) was not inhibited by Brefeldin A reduction of Stx5. Instead, the inhibition of fibril formation by monensin suggested that the vesicles might share the properties of LAMP1-containing lysosome-like compartments. The involvement of compartments containing PCI and lysosomal proteins, such as LAMP1, in fibril generation was unexpected. Non-classical routes of secretion have been described for various cargoes; however, these typically carry proteins without signal peptides (for review see ( Kim *et al.*, 2018)). Some Golgi-bypassing mechanisms have been described, for example, ER-targeted cargoes such as alpha integrins, the thrombopoietin receptor, and mutant forms of CFTR (reviewed by ( Kim *et al.*, 2018)). The term ‘secretory lysosome’ has been used to describe organelles that function in both the degradation and storage of secretory proteins (reviewed by ( Blott & Griffiths, 2002)). Secretory lysosomes are found in T cells, natural killer cells, mast cells, and macrophages. Natural killer cells release toxic proteins by secretory lysosomes (reviewed by ( van der Sluijs *et al.*, 2013), and in cancer, secretory lysosomes can be a temporary store of immune checkpoint proteins (reviewed by ( Wang *et al.*, 2020)). Wounding of fibroblasts leads to the presentation of lysosomes to the cell periphery, which is important for the repair of the cell membrane ( Reddy *et al.*, 2001). This presentation of lysosomes to the cell periphery has also been observed during the differentiation of osteoblasts at times of enhanced collagen production ( Beck *et al.*, 2001; Nabavi *et al.*, 2008). Thus, beyond its well-documented role in protein degradation and recycling, lysosomal systems play a role in exocytosis for cellular clearance and cell-cell communication (reviewed by ( Buratta *et al.*, 2020). In the context of collagen trafficking, lysosomes have typically been viewed as degradative compartments via the autophagy pathway ( Gorrell *et al.*, 2021). We propose that fibroblasts use lysosomes or lysosome-like compartments to store and concentrate procollagen and collagen. Indeed, imaging approaches using Dendra2 showed the existence of the pool in the presence of active PCI synthesis, and SILAC studies confirmed long-lived intracellular PCI with t½ ~ 68 h. The lysosome is an ideal storage compartment for type I collagen because its acidic pH prevents spontaneous fibril formation ( Harris & Reiber, 2007) and is a stable environment for the collagen triple helix, which is highly resistant to degradation by proteinases such as pepsin. This storage function represents a state of preparedness in the event of tissue damage or wounding. The rate of appearance of collagen in the lysosome compartment is slower than that through the ER ( Leblond, 1989) and others have demonstrated the accumulation of collagen within the lysosome when applying high concentrations of inhibitors ( Forrester *et al.*, 2019). Recent studies following the trafficking of type I and II collagens in the presence of lysosomal inhibitors also suggest that collagen is trafficked to lysosomes ( Forrester *et al.*, 2019). Evidence from previous studies suggests that not all collagen is secreted rapidly; labelling of newly synthesized collagen in rat fibroblasts appears in the lysosome at a slower rate than in the Golgi ( Leblond, 1989). Together, these studies suggest that collagen may utilize this route during its exit from the cells. In the present study, the importance of the lysosomal compartment in collagen fibril formation was demonstrated using lysosomal inhibitors and in fibroblasts derived from patients with lysosomal storage disorders, where lysosomal function is disrupted due to improper processing of glycoproteins. Importantly, these fibroblasts continued to secrete type-I collagen in the absence of fibril formation, which demonstrates the distinction between collagen secretion and fibril assembly. This defect in the ability to assemble an effective matrix warrants further investigation to understand its contribution to skeletal and connective tissue malformations associated with MPS. 

Our experiments using a bioreactor showed that fibril nucleation (the start of a new fibril) and fibril propagation (lengthening of an existing fibril) are separate processes. Under static conditions, fibrils nucleated on the plasma membrane and subsequently extended in length. However, when the cells were placed under flow, the number of fibrils on the cell surfaces did not change, whereas the fibrils were shorter. The reduction in fibril length could be attributed to either a reduction in soluble collagen in the culture medium or to a cellular response to flow. In the cell cultures used here, fibrils occurred exclusively on the underside of the cell, where a microenvironment most likely exists to promote fibril elongation. However, it is unclear how flow across the apical surface changes the microenvironment at the basal surface. Therefore, a feedback mechanism might exist to divert the secretion of collagen from the basal surface to the apical surface, depending on how the cell senses flow. The pathway of rapid collagen egress from the cell is clearly dependent upon traffic from the ER to the Golgi, as described elsewhere ( Bonfanti *et al.*, 1998), therefore it remains to be understood how flow across the cell surface might affect ER-to-Golgi transport. 

In conclusion, the use of CRISPR/Cas9 to tag procollagen, combined with absolute quantitation of collagen and high-resolution microscopy, has allowed the visualization of collagen fibril formation by living cells. The identification of the separate handling of collagen for fibril nucleation and propagation provides an explanation for how cells might regulate the number and growth of fibrils. Our study also provides extensive data on fibril formation *in vitro* and electron microscopy of fibrils *in vivo* into a unified model of cell-regulated nucleation-and-propagation to explain how cells orchestrate tissue structure via the control of collagen fibril assembly.

## Data Availability

ProteomeXchange: Consortium via the PRIDE partner repository: mass spectrometry proteomics data. Accession number PXD036794; https://dx.doi.org/10.6019/PXD036794 ( Perez-Riverol *et al.*, 2022). **Figshare:** Pickard (2025). Collagen fibril formation at the plasma membrane occurs independently from collagen secretion. Extended Data and underlying data [Dataset]. figshare. https://doi.org/10.6084/M9.FIGSHARE.29207657 ( Pickard, 2025). The project contains the following underlying and extended data: Figures 1–8 underlying data Extended data 1: Nluc-PCI and Dendra2-PCI generation Extended Data 2: Intracellular collagen turnover over 2 days. Extended Data 3: Deposition of Dendra2 tagged type I collagen into the matrix. Extended data 4: Rapid release of intracellular collagen at the time of fibril formation. Extended Data 5: Dendra2 positive fibrils detected on the basal surface of cells. Extended Data 6: Photoswitched Dendra2 positive fibrils detected on the basal surface of cells. Extended data 7: Endocytosed collagen contributes to fibril assembly. Extended data 8: Non-conventional PCI trafficking at sites of fibril nucleation *.* Extended data 9: Quantitative real time PCR of syntaxin-5 knock down. Extended data 10: Proteins identified in lysosomal fractions by mass spectrometry Extended data 11: Dendra2-PCI colocalizes with ER and lysosomal-like compartments Extended data 12: Confirmation of mutations in MPS I and MPS IIIA fibroblasts Extended Data 13: Photoswitched Dendra2 positive fibrils detected on the basal surface of cells. Extended Data 14: Fibripositors are regularly contacted by endoplasmic reticulum and electron dense vesicles. Extended Data 15: The Golgi apparatus does not contact fibripositors. Extended data 16: Primers used for generating repair templates and BFP tagged HSP47. Extended data 17: Primer sequences used to create split nanoluciferase knock-in repair templates. Extended data 18: Primers used for real time PCR quantification of transcripts. Data are available under the terms of the Creative Commons Attribution 4.0 International license (CC-BY 4.0).
