# Supplementary material for: Collagen fibril formation at the plasma membrane occurs independently from collagen secretion
Source: Wellcome Open Res. 2025 Aug 29;10:468. [Version 1] doi: 10.12688/wellcomeopenres.23776.1 (PMC12595305; doi:10.12688/wellcomeopenres.23776.1)
Supplement: Peer review history

# Reviewer report: Reviewer response for version 1

**DOI:** 10.21956/wellcomeopenres.26224.r133099

**Published:** 2025-11-08

**Authors:** Alexandra Naba

**Affiliations:** 1 University of Illinois Chicago, Chicago, USA

**Overall assessment**

 In this manuscript, Pickard and colleagues tackle a fundamental question in the field of collagen and extracellular matrix (ECM) biology: how are collagen fibrils assembled by cells? 

 Understanding these mechanisms can lead to important breakthroughs in biomedicine as they can offer therapeutic solutions to the dozens of pathologies, or “collagenopathies”, caused by mutations in collagens, and to pathologies characterized by excessive collagen deposition, such as fibrosis and cancer. Understanding these mechanisms can also help design more effective tissue repair and regeneration strategies, as well as combat aging. This study is thus highly significant, and the amount of work performed is undeniable. However, the description of the methods and the findings, as well as parts of the introduction, is elusive, and the connection between the different subsections of the Results section is not always obvious. In addition, the manuscript appears to have been written for readers with extensive prior knowledge of the collagen biosynthetic pathway and the mechanisms of collagen assembly, as well as expertise in SILAC-based proteomics, CRISPR-mediated knock-in, and sophisticated imaging. As a result, readers may find it difficult to evaluate the rigor of the analyses presented. I encourage the authors to thoroughly revise their manuscript to provide more details on the experimental design and make it more accessible to a broader audience.

**Major comments on Results: **

- Dendra2 constructs: Can the authors comment on the cleavage of the Dendra2 fluorescent protein upon processing of the N-propeptide of the a2 chain of collagen I extracellularly? Could the schematic of the constructs be enhanced to depict the processing of PCI?

 

- It is unclear how the authors concluded that “fibroblasts secrete approximately 100,000 procollagen molecules per hour”.

 

- The authors imply that, in addition to the pool of procollagen I present in the ER and transiting through the Golgi, a pool remains intracellular for up to 48h. In which intracellular compartment does this pool reside? Is the entirety of this pool destined for secretion? What would be the role of this pool?

 

- SILAC MS:
  - The use of “half-life” might be misleading since intracellular collagens do not decay (at least not all) but are rather secreted. Perhaps the authors could refer to it as the “intracellular half-life”? Also, in their sample preparation. How can the authors distinguish between the intracellular and extracellular (pericellular) pools?
  - It would be interesting to compare the half-life of collagen I  with the half-life of proteins important for the collagen biosynthetic pathway (prolyl hydroxylases, lysyl hydroxylases,  proteins involved in collagen trafficking through the Golgi, collagen receptors at the cell surface, etc). It would also be interesting to compare it to the half-life of other ECM proteins, such as fibronectin. Could the authors extract the data from their SILAC-MS dataset?
  - Can the authors help readers interpret biologically the meaning of a half-life of 69.8 hours determined for procollagen I?
  - Can the authors expand on the difference in the half-life calculated using imaging and mass spectrometric measurements?

 

- Extended Data 6 is difficult to interpret and does not definitively show the association of fibrils with the basal of the cells. An xz projection (the one provided in Figure 3 is also difficult to read) would depict this better, but the definitive answer will be provided by EM. In addition, what do the authors imply when stating that “Dendra2-positive fibrils were deposited on the basal cell surface, near but distinct from the BFP signal” and that “close proximity of the ER with the extracellular collagen fibril, with the ER arranged aligned to the forming collagen fibrils, suggesting that these sites may contribute to rapid secretion”? Are the authors suggesting that there is direct secretion from the ER to the plasma membrane/pericellular space? How about Golgi trafficking?

 

- Can the authors provide clarification as to whether the secretion of collagen is polarized in their cellular system? Or is it only the assembly that is polarized?

 

- It would be very helpful to have a model clearly schematizing the different steps in collagen I secretion and the new findings of the manuscript (secretion of newly synthesized molecules, distinct intracellular pools, polarization of assembly, source of molecules used for nucleation vs elongation, etc.).

**Major comments on Materials and Methods**

- SILAC MS sample preparation: Can the authors provide evidence (immunoblot) that the lysis buffer used fully extracts the pericellular collagen being assembled into fibrils at the cell surface?

 

- MS database search: Collagens are known to undergo hydroxylation on the amino acids lysine and proline, and prior research has shown that allowing for these post-translational modifications as “variable” modifications in the database search significantly increases the number of collagen PSMs and allows for more accurate quantification (Basak and Vanacore; Naba and Hynes); however, the authors omitted to include these modifications in their search parameters. In the context of this study, the authors could also leverage ratios of hydroxylated vs non-hydroxylated procollagen I and compare the pools of newly synthesized procollagen I vs the pool of procollagen I molecules that are transiting to the Golgi, where they undergo these PTMs, vs the pool of secreted collagens. Can the authors comment on this?

 

- What standards were used for the hydroxylproline assay?

 

- Although I am not an expert in high-resolution microscopy, it is my understanding that the airy scan mode on a Zeiss 880 typically achieves a resolution of ~120-140nm in the lateral dimension and of ~350-400nm in the axial dimension, so it cannot resolve the periodicity of the collagen fibers. However, the manuscript suggests that the airy scan measurements were used to determine fiber periodicity. Can the authors please clarify this?

 

- It would be helpful if the authors could indicate how the mouse models used in the study can be obtained (source, strain number).

 

- Since antibody concentrations can vary from lot to lot, rather than listing the dilution factors, it would be preferable to indicate the final concentration at which each antibody was used.

**Other comments**

- I would encourage the authors to review the manuscript thoroughly to ensure that they use consistent and precise wording to describe molecules and processes. For example, it seems a bit familiar to write “Similar to the effect of Dendra2 tagging on *type I* secretion”.

 

- Some results are stated in a very definitive manner, but the data underlying them are not always definitive. For example, the authors state that they  “[have] established that fibril nucleation is under cellular control”; to claim this, one would need to abrogate cellular control, ideally in a few different ways, and show that nucleation is unable to proceed, which was not done.

Is the work clearly and accurately presented and does it cite the current literature?

Partly

If applicable, is the statistical analysis and its interpretation appropriate?

Yes

Are all the source data underlying the results available to ensure full reproducibility?

Yes

Is the study design appropriate and is the work technically sound?

Partly

Are the conclusions drawn adequately supported by the results?

Partly

Are sufficient details of methods and analysis provided to allow replication by others?

No

Reviewer Expertise:

Extracellular Matrix Biology; ECM Proteomics, Tumor Microenvironment, Cell Biology

I confirm that I have read this submission and believe that I have an appropriate level of expertise to confirm that it is of an acceptable scientific standard, however I have significant reservations, as outlined above.

# Reviewer report: Reviewer response for version 1

**DOI:** 10.21956/wellcomeopenres.26224.r133101

**Published:** 2025-09-27

**Authors:** Tilo Pompe

**Affiliations:** 1 Leipzig University, Leipzig, Germany

The authors present a large number of new findings on the process of cell-driven formation of collagen I fibrils. This work represents an interesting topic as the exact process of collagen I fibril formation in live cell systems is currently not fully understood. Overall a wealth of advanced methods was used to reveal and validate details of cell-driven collagen synthesis and fibril formation. However, a revision of the provided presentation is needed to allow readers to follow the approach of investigations and the line of argumentation. The following detailed comments should be observed when revising the manuscript.

 Introduction

 ==========

 The introduction is very short and difficult to follow.

 1. In the beginning collagen is introduced, but, the wealth of collagen subtypes is not mentioned and the specific focus of the work on fibrillar collagen 1 is not introduced.

 2. At the end of the introductory section (last 5 sentences on goal and content of the work) the text is rather hard to follow and loosely connected in content flow. It should be rewritten and smoothed.

 Methods

 =======

 Overall the methods section is frequently written in a sloppy manner and should be carefully revised.

 E.g.

 - There are various typos, especially for units and brackets.

 - There is an inconsistent way of using abbreviations, e.g. "transmission electron microscopy" vs. "SEM" vs. "ImmunoEM"

 - "Photoswitching" details are mentioned, but, it is not clearly state of what, it is probably Dendra2.

 Results

 ======

 1. It is not clear (from Methods or Results section), how the following result/statement is determined: "Our quantification analysis revealed that fibroblasts secrete approximately 100,000 procollagen molecules per hour, ..."

 2. Strange wording is used: "synthetic capacity" , better "capacity of synthesis"

 3. It is not clearly described in the section on tracking of procollagen how the half-life of approx. 24 h is determined. It is probably from SILAC experiments, but, this does not come out here. It should be also made clear what is meant by "half-life" in the results section at this position, localization within cell, degradation, or ?. This issue is firstly mentioned in the discussion section.

 4. It is not easy to understand, why no Dendra2 fibrils appear within 36 h of imaging. How is the time point of "onset imaging" is correlated to any cellular function or induction of Dendra2 collagen / fibrils?

 5. How is the periodicity of collagen fibrils determined in Fig.2B? There is not description in methods or results section on this fact. From which fibril(s) in image 2C such an analysis is performed? From a brief look the thin fibril at left has a different periodicity and structure than the thicker fibril on the right.

 Discussion

 ========

 1. It is not clear, how the following statement  is determined and from which data: "but that collagen fibril assembly occurs at the plasma membrane every 24 h via an intracellular pool." There should be an appropriate description and presentation in the results section.

 Figures

 ======

 Overall many figures (and captions) are prepared in a sloppy manner and have to be revised.

 This similarly concerns figures in the extended data.

 E.g.

 - Figure 1: usage and capitalization of units in diagram are strange and inconsistent with other figures and common approaches (e.g. mins, Hours, hrs, ...); size of scale bars is missing frequently

 - Figure 2: size of scale bar is missing for 2B; size of zoom image is missing

 - Figure 5: scale bars or / and size of scale bars are missing

 - Figure 6: low contrast in image 6D + 6E

 - Figure 7B: scale bar is missing, caption: meaning of colored arrows is missing and mentioned "red box" is not seen in the figure

Is the work clearly and accurately presented and does it cite the current literature?

Partly

If applicable, is the statistical analysis and its interpretation appropriate?

Yes

Are all the source data underlying the results available to ensure full reproducibility?

Partly

Is the study design appropriate and is the work technically sound?

Yes

Are the conclusions drawn adequately supported by the results?

Partly

Are sufficient details of methods and analysis provided to allow replication by others?

Partly

Reviewer Expertise:

biomaterials, extracellular matrix engineering and analysis, optical microscopy and image analysis, extracellular matrix biology

I confirm that I have read this submission and believe that I have an appropriate level of expertise to confirm that it is of an acceptable scientific standard, however I have significant reservations, as outlined above.

# Reviewer report: Reviewer response for version 1

**DOI:** 10.21956/wellcomeopenres.26224.r133104

**Published:** 2025-09-26

**Authors:** Mario Raspanti

**Affiliations:** 1 Insubria University, Varese, Italy

The manuscript is of excellent quality and I have nothing to object to its scientific content. The Introduction is concise but satisfactory, the Methods section is probably the most complete I have ever seen and the Results deploys an outstanding array of different techniques, well summarized in the Discussion.

 Just a few points emerge here and there:

 - The Methods section includes a "Serial Block Face SEM" section that is not used in this research.

 - In Figure 1 the D and E letters seem to have been swapped. The white arrows in F are not mentioned  in the caption.

 - In Figure 2 it would be useful to specify that the AFM picture spans 2x2 micron.

 - Near the end of the Results there is a typo: MPSI IIA appears instead of MPSI IIIA.

 - The Results convincingly show that the cells control the fibrils formation near their basal portion, but that they do so by "tethering one end of the fibril to the plasma membrane" as reported in the very beginning of the Discussion is not so evident.

 - The Supplementary Data repository contains multiple (previous?) versions of the same data. For instance, the picture in "Pickard-Extended data 12.pptx" also appears at the end of "Pickard-Extended data.pptx" (no number) where it is listed as S7.

Is the work clearly and accurately presented and does it cite the current literature?

Yes

If applicable, is the statistical analysis and its interpretation appropriate?

I cannot comment. A qualified statistician is required.

Are all the source data underlying the results available to ensure full reproducibility?

Yes

Is the study design appropriate and is the work technically sound?

Yes

Are the conclusions drawn adequately supported by the results?

Yes

Are sufficient details of methods and analysis provided to allow replication by others?

Yes

Reviewer Expertise:

Electron microscopy; extracellular matrix

I confirm that I have read this submission and believe that I have an appropriate level of expertise to confirm that it is of an acceptable scientific standard.
